# Recent Advances of Fe(III)/Fe(II)-MPNs in Biomedical Applications

**DOI:** 10.3390/pharmaceutics15051323

**Published:** 2023-04-23

**Authors:** Weipeng Chen, Miao Liu, Hanping Yang, Alireza Nezamzadeh-Ejhieh, Chengyu Lu, Ying Pan, Jianqiang Liu, Zhi Bai

**Affiliations:** 1 The First Dongguan Affiliated Hospital, Guangdong Medical University, Dongguan 523700, China; 2 Guangdong Provincial Key Laboratory of Research and Development of Natural Drugs, and School of Pharmacy, Guangdong Medical University, Guangdong Medical University Key Laboratory of Research and Development of New Medical Materials, Dongguan 523808, China; 3 Chemistry Department, Shahreza Branch, Islamic Azad University, Shahreza 311-86145, Iran; arnezamzadeh@iaush.ac.ir; 4 Affiliated Hospital of Guangdong Medical University, Zhanjiang 524013, China

**Keywords:** Fe-based MPN, tumor treatment, CDT, PTT, Fenton reaction

## Abstract

Metal–phenolic networks (MPNs) are a new type of nanomaterial self-assembled by metal ions and polyphenols that have been developed rapidly in recent decades. They have been widely investigated, in the biomedical field, for their environmental friendliness, high quality, good bio-adhesiveness, and bio-compatibility, playing a crucial role in tumor treatment. As the most common subclass of the MPNs family, Fe-based MPNs are most frequently used in chemodynamic therapy (CDT) and phototherapy (PTT), where they are often used as nanocoatings to encapsulate drugs, as well as good Fenton reagents and photosensitizers to improve tumor therapeutic efficiency substantially. In this review, strategies for preparing various types of Fe-based MPNs are first summarized. We highlight the advantages of Fe-based MPNs under the different species of polyphenol ligands for their application in tumor treatments. Finally, some current problems and challenges of Fe-based MPNs, along with a future perspective on biomedical applications, are discussed.

## 1. Introduction

Cancer is one of the most serious threats to human health [1]. In 2022, the latest global cancer burden data released by the International Agency for Research on Cancer (IARC) of the World Health Organization (WHO) showed that 19.29 million new cancer cases occurred worldwide in 2020 [2]. To date, there have been various cancer treatment options. The optimization and improvement of tumor treatment is a hot topic of interest for the scientific community in recent years. They enhance the therapeutic targeting and efficacy of drugs by coating the surface of drugs with materials of nano-films or nano-frameworks to reduce drug toxicity to the human body. Chemodynamic therapy (CDT) [3,4] and phototherapy (PTT) [5,6] are two green and highly effective tumor treatments in biomedicine that scientists optimally consider.

In the last decade, a coating material called metal–phenolic networks (MPNs) has gradually entered the research field of therapeutics, providing a novel research strategy for antibacterial therapy [7] and antitumor therapy [8]. MPNs are three-dimensional stable supramolecular organic–inorganic hybrid networks, formed based on coordination using different metal ions with polyphenolic ligands, similar to crystalline porous coordination polymers (PCPs) and metal-organic framework (MOFs) [9] type materials. Natural polyphenols, such as tannic acid (TA) [10], gallic acid (GA) [11], and epigallocatechin gallate (EGCG), have been reported to be the phenolic ligand regulars used for the preparation of MPNs. In contrast, the successful coordination of metal ions, such as Fe^3+^, Cu^2+^, Zn^2+^, Mn^2+^, Ni^2+^, and Co^3+^, with polyphenols to form MPNs has been widely used in the study of the surface modification and therapeutic environment modulation of various materials [12,13].

Despite the rapid developments of the MPNs in several research fields, their applications in tumor treatment have created considerable advantages. Compared to the MOF-type nanomaterials, MPNs with excellent safety and non-toxicity, as well as faster and cost-effective preparation methods, are also more environmentally friendly and readily available materials [14,15]. MOFs tend to be prepared under more stringent conditions, with longer preparation times and higher costs. Some organic materials, such as liposomes, polymers, and dendrimers, are usually biodegradable, have low toxicity, and are chemically modifiable, but they still suffer from deficiencies in controlling the targeted release of drugs as nanomaterials [16]. Compared with common metallic nanomaterials and polyphenols, MPNs have many highlighted advantages. Firstly, the phenolic hydroxyl groups of organic ligands (polyphenols) provide a high affinity with good adhesion to the surface of various materials. MPNs are capable of effectively adsorbing and encapsulating any topological forms of substances, giving them new functions [17,18,19]. Not only that, MPNs always have good pH responsiveness. Generally, they are stable at pH 7–8, making long-term blood circulation possible under physiological conditions.

On the contrary, the interaction between polyphenols and metal ions readily dissociates in low pH environments, providing MPNs with a pH-adjusted metal ion release [20,21]. The specific acidic microenvironment at tumor sites helps MPNs to target drug release in tumors compared to normal tissues [22], thereby reducing off-target adverse effects and allowing them to perform different functions in different environments. Besides, most polyphenols have strong antioxidant properties and can effectively scavenge metal ion-induced excess reactive oxygen species (ROS) to maintain the homeostasis of the local microenvironment. Equally important, the synergistic effect of polyphenols and metal ions in MPNs can optimize the tumor therapeutic environment, improve the antibacterial activity, and enhance the material’s biocompatibility [23]. Therefore, various superior properties have led to the frequent use of MPNs as drug carriers for hollow capsules [20], surface coatings for materials [24], pH-responsive agents for tumor microenvironments (TME) [21], and antibacterial composite nanoparticles [24]. Since it was first reported in 2013 [14], MPN coatings have become convenient functional nanocoatings covered with different substrate arrays, impacting the application of engineered materials and clinical therapeutic aspects.

Fe is one of the common essential trace elements in the human body. It is involved in normal physiological activities. It is more closely related to immunity, leading to increased resistance against infection in the body [25], making it a prime candidate among therapeutic materials. Numerous studies on Fe-based nanomaterials have been reported, especially Fe-based nanoparticles. Among them, Zhong et al. summarized the progress of biomedical research on nanomaterials of Fe-based MIL in the MOF family. Recently, they described the study of Fe-based MIL in drug delivery systems (DDSs), and they emphasized that the biosafety of nanomaterials is an essential indicator for medical research [26]. It is easy to see that Fe-based nanomaterials have great research significance and promising applications.

**Figure 1:**
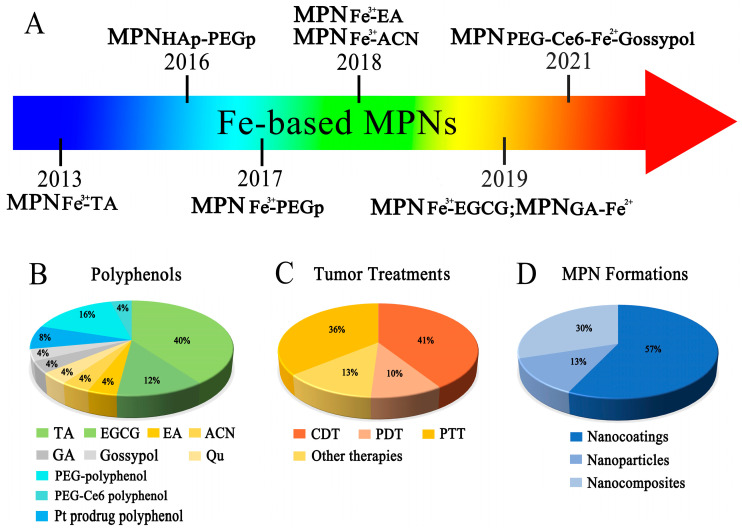
(**A**) Milestones in developing Fe-based MPNs in tumor treatment [14,33,34,35,36,37,38,39]. Pie charts (**B**) show the percentage of used polyphenols by Fe-based MPNs, (**C**) different therapies by Fe-based MPNs in tumor treatments, and (**D**) the formation of Fe-based MPNs. The above data are according to Table 1 and Table 2.

**Scheme 1:**
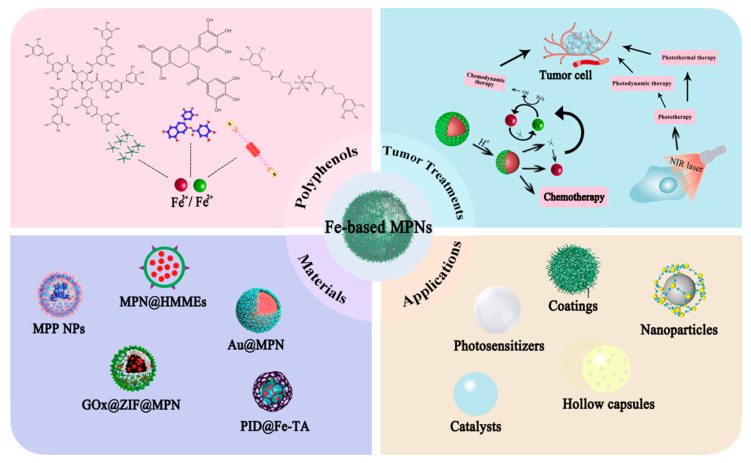
Scheme of polyphenol use and the therapeutic mechanism of Fe-based MPNs with representative materials and applications.

Moreover, Fe-based MPNs are the most common metal–phenolic network materials in tumor treatment. Due to being frequently used as surface coating modifications for drugs, researchers have developed various pH-responsive nanosystems based on their adhesion and plasticity. Due to easy leakage, surface modifications can protect drug molecules from tissue toxicity [27,28]. In addition, they can be used as nanoparticles and nanocomplexes that are released as free radical scavengers or catalysts for synergistic therapy when they reach the tumor site, under co-encapsulation with the drug, to achieve synergistic therapeutic effects. Compared to other Fe-based nanomaterials, the Fe-based MPNs would be superior Fenton reaction catalysts, releasing Fe^3+^/Fe^2+^ mediated autocatalytic Fenton reaction under TME to produce highly toxic hydroxyl radicals (•OH) that effectively help the drug to kill tumor cells in CDT [29,30]. The application of PTT can be divided into two parts. Fe-based MPNs in PTT can transport drugs to tumor sites through coatings, hollow capsules, etc. [31]. Still, due to their excellent photothermal conversion efficiency, they are often used as photothermal agents to kill tumor cells by heating up at the tumor site under laser irradiation. They are also used as photosensitizers in PDT that are often synergistic with the Fenton reaction to substantially enhance PTT’s therapeutic effect and effectively kill tumor cells while protecting normal cells [32]. In the following, we will summarize Fe-based MPN materials (Scheme 1), under different preparation strategies based on polyphenols, with their applications in CDT and PTT (Figure 1). In addition, some analyses and perspectives on the problems in the treatment process will be presented.

## 2. Characteristics and Designing Strategies for Fe-Based MPNs

It is well known that Fe is one of the essential trace elements for biological survival [25], and its ions can spontaneously react with hydrogen peroxide (H_2_O_2_) in a Fenton manner during normal physiological processes in vivo under certain conditions, leading to intracellular production of •OH. Some other metal ions, such as Al^3+^, Cu^2+^, Mn^2+^, etc., have shown a strong Fenton reaction or Fenton-like activity [57,58,59], but Fe^3+^/Fe^2+^ is the most widely used in practice. Fe ions are characterized by high connectivity and low biotoxicity, making them among the most widely used components in Fenton reactions in tumor treatment. The inherent properties of polyphenolic compounds, including metal chelating properties, pH reactivity, redox potential, free radical scavenging, polymerization, and photo-absorption, enable their ability to develop MPN materials and prepare various structural substrates [60,61]. Polyphenolic compounds are classified into natural and artificial polyphenols. Here, we will focus on the characteristics and design strategies of some Fe-based polyphenolic complexes and MPNs formed by them.

### 2.1. Fe with TA

**Figure 2:**
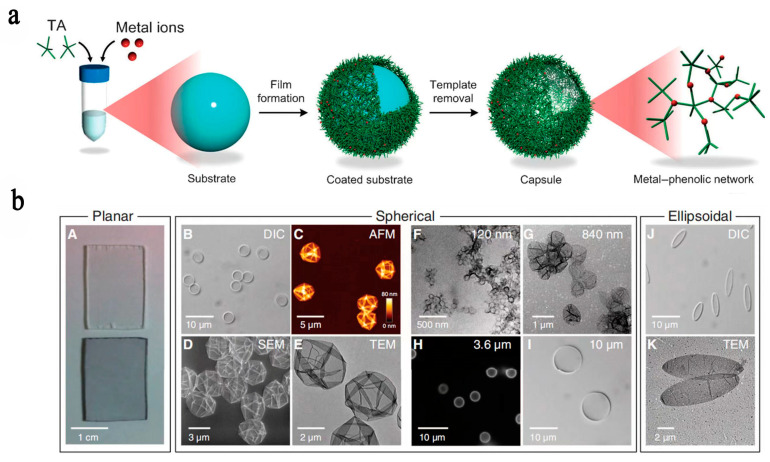
(**a**) Assembly of MPN capsules from various metals. Assembly of TA and metal ions to form an MPN film on a particulate template, followed by the subsequent formation of an MPN capsule. Reprinted with permission from Ref. [12] (copyright 2014, Wiley). (**b**) Fe^III^–TA films were prepared on PS substrates with various shapes (planar, spherical, and ellipsoidal) and sizes (D = 120 nm to 10 mm). (**A**) Photograph of PS slides before (top) and after (bottom) Fe^III^-TA coating. (**B**–**K**) Microscopy images of Fe^III^-TA capsules: DIC images (**B**,**I**,**J**), AFM images (**C**), TEM images (**E**–**G**,**K**), SEM image (**D**), and fluorescence microscopy image (**H**). It is reprinted with permission from Ref. [14] (copyright 2013, Ejima).

Tannic acid (TA), one of the common natural polyphenols, can chelate various metal ions due to its phenolic hydroxyl-rich structure, and it can form metal–TA complexes with pH-sensitive decomposition properties via dialkyl groups. This outstanding property makes it an ideal candidate for linking metal ions and catalyzing reactions [62,63]. Due to its antioxidant properties, TA can act as a good scavenger of free radicals and ROS; not only that, TA has also received much attention for its anticancer activity. In 2013, Ejima et al. successfully synthesized TA–Fe^3+^ films, for the first time, by a one-step assembly of Fe^3+^ ions with natural polyphenol TA. Its excellent bio-tunable physicochemical properties with the easy assembly process, low cost, and scalability, coupled with pH responsiveness and negligible cytotoxicity, make it a highly potential material for biomedical applications [14] (Figure 2). It is the pioneer of various MPN materials, so much so that TA–Fe^3+^ films have become the most commonly used coating material in MPNs.

### 2.2. Fe with GA

**Figure 3:**
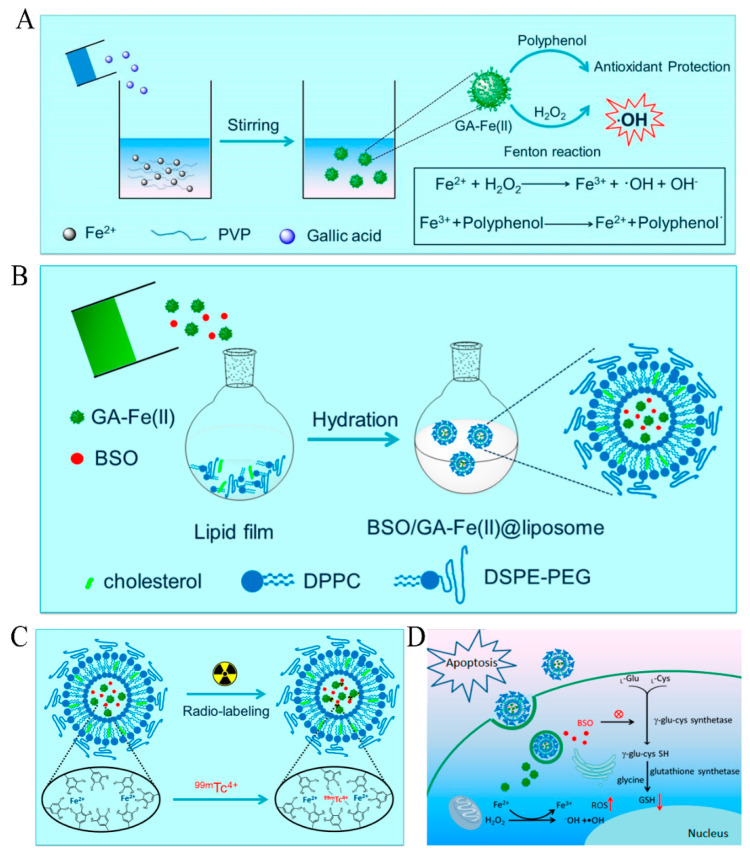
(**A**) Schematic illustration of the synthesis and functions of GA–Fe(II) nanocomplexes. (**B**) A scheme showing the preparation of BSO/GA–Fe(II)@liposome. (**C**) A scheme showing ^99m^Tc-labeled nanoparticles. (**D**) Schematic illustration of the amplified oxidative stress via the effective •OH production and GSH depletion to induce cell death. Reprinted with permission from Ref. [33] (copyright 2019, Dong).

Amplifying intracellular oxidative stress is an effective strategy to induce cancer cell death. Gallic acid exhibits strong free radical scavenging ability and shows antioxidant activity to maintain an endogenous antioxidant defense system and prevent lipid peroxidation [62]. When ligated with Fe^3+^ or Fe^2+^, it exhibits a mild pro-oxidant effect. Dong et al. prepared a unique ultrasmall nanocomplex (GA-Fe^2+^) by self-assembly, which was not coated on the material’s surface as MPN but was encapsulated in liposomes to act as an efficient Fenton reaction catalyst in therapy. Enabling the sustained H_2_O_2_ conversion to highly cytotoxic •OH greatly amplifies intracellular oxidative stress [33] (Figure 3). It is easy to see that the strategy of encapsulating and enhancing intracellular oxidative stress by using MPNs as nanoparticles certainly opens up a new design idea for tumor treatment.

### 2.3. Fe with EGCG

**Figure 4:**
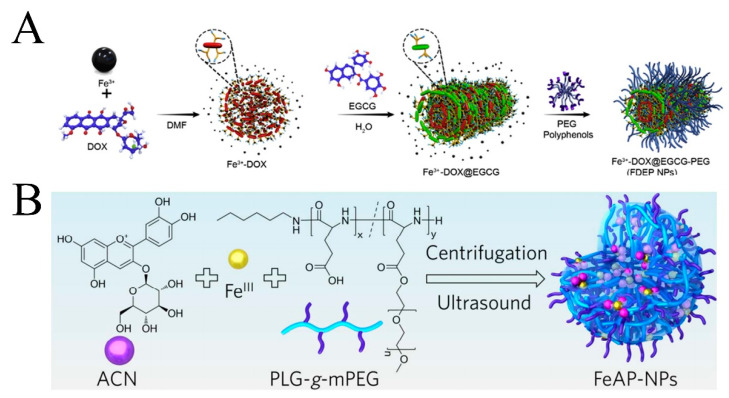
(**A**) Formulation and characterization of nanoparticles and the proposed mechanism of enhanced anticancer effects. Reprinted with permission from Ref. [34] (copyright 2019, Shan). (**B**) Schematic illustration for the preparation and structure of FeAP-NPs. Reprinted with permission from Ref. [35] (copyright 2018, Xu).

Epigallocatechin gallate has antioxidant, anti-inflammatory, and anticancer benefits [62], and it is the most abundant and unique compound among tea polyphenols [64]. As a rapidly emerging star among natural active ingredients, it can effectively and specifically inhibit the tumor proteasome activity associated with the induction of apoptosis in tumor cells. It is a natural active ingredient with potent anticancer effects caused by promoting the intracellularization of platinum-based drugs, DNA–Pt interactions, and, ultimately, apoptosis [65,66,67]. Several reviews have reported MPN based on the formation of Fe ions with EGCG, some as nanoparticles sequestered with the material together, and some forming surface coatings with the help of other compounds. In a representative case, Shan et al. designed an MPN formed by the formation of EGCG and polyethylene glycol (PEG) polyphenols assembled with Fe^3+^ by self-assembly, taking advantage of the effective anticancer and coordination properties of EGCG with metal ions [34] (Figure 4A). The therapeutic nanoplatform integrating DOX and EGCG has efficient tumor delivery and negligible cardiotoxicity, which provides great potential for MPNs in clinical cancer therapy.

### 2.4. Fe with ACN

Combining natural polyphenols with copolymers is a superior strategy to ensure low therapeutic toxicity and facilitate diagnostic tumor imaging. Anthocyanins (ACN) are natural polyphenols derived from blue honeysuckle, a component with active therapeutic and non-toxic properties in ophthalmology, inflammation, and coronary artery disease [68]. Xu et al. combined ACN and Fe^3+^ for the first time to form MPN nanoparticles, paired with a PLG–g–mPEG copolymer, to improve nanoparticle versatility and safety (Figure 4B). It showed good biocompatibility and physiological stability in tumor treatment with good dual PA/MR imaging properties. Not only is it highly promising for the treatment of liver and kidney applications but it also provides a new MPN design strategy for oncology treatment [35].

### 2.5. Fe with EA

Ellagic acid (EA), a natural polyphenol widely found in various soft fruits, nuts, and other plant tissues, shows similar physicochemical properties to other natural polyphenols [69]. EA has been shown to exhibit significant inhibitory effects on various cancers, including breast, skin, and lung. Inspired by the coordinated metal–TA complexes in hollow capsules studied by Caruso’s team, Zhao et al. extended the coordination theory between metal ions and the catechol group to EA. As a result, MPN nanoparticles were prepared, for the first time, by combining EA with Fe^3+^ in a facile green synthesis method, which imparts high-quality photothermal effects and provides a new imaging-guided therapeutic system for tumor ablation in combination with the magnetic resonance capability of Fe^3+^ [36].

### 2.6. Fe with Qu

Based on previous nanodrug studies, many strategies have good tumor-damaging effects. However, the rapid clearance of small-size nanoparticles (<6 nm) through the renal system is contradicted by the larger-size nanoparticles that are more favorable for inducing enhanced permeability and retention (EPR) effect-mediated tumor accumulation. Developing new biodegradable nanomaterials with dynamic demolition capabilities became the preferred strategy of Yang et al. to overcome this dilemma between renal clearance and EPR-mediated tumor accumulation. Inspired by this, they developed a multifunctional Qu–Fe^II^P nanoparticle, a nanomaterial based on quercetin (Qu), Fe^2+^, and polyvinylpyrrolidone (PVP). Qu is a natural polyphenol widely found in vegetables and fruit peels with antioxidant and anti-cancer properties. This nanodrug presents pharmacological activity against cancer in vivo and provides an opportunity for disassembly and elimination [53,70]. It is the first MPN nanomaterial that combines Qu with Fe^2+^ in the application of tumor treatment. This is another new achievement of natural polyphenols’ application in tumor treatment.

### 2.7. Fe with PEG-Polyphenol

**Figure 5:**
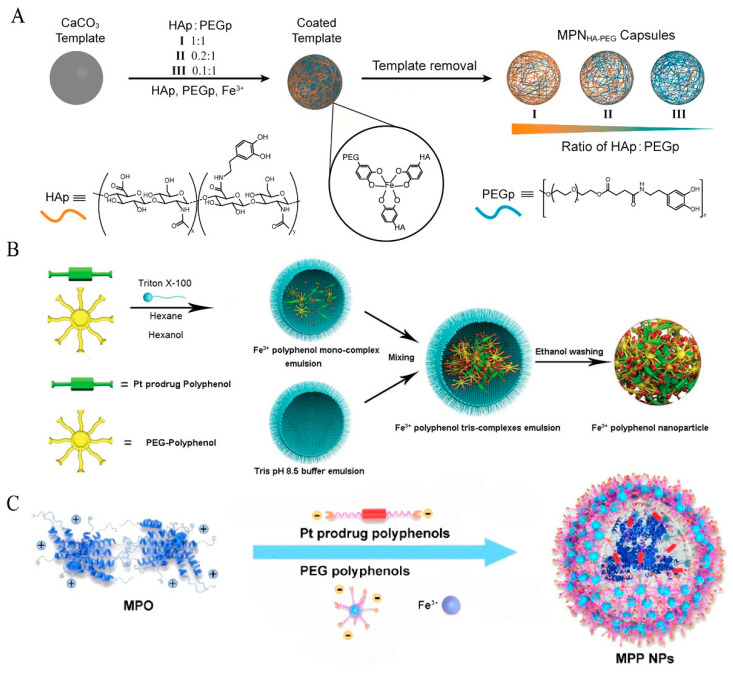
(**A**) Illustrates the metal–phenolic assembly of MPNHA-PEG capsules. MPNHA-PEG capsules with three different HA/PEG ratios were prepared by adding HA-polyphenol (HAp, orange) and PEG-polyphenol (PEGp, blue), at three different ratios, to a suspension of CaCO_3_ particles, followed by adding Fe^III^, increasing the pH, and dissolving the templates. Reprinted with permission from Ref. [37] (copyright 2016, Ju). (**B**) Schematic of the PtP NP self-assembly process. Reprinted with permission from Ref. [38] (copyright 2017, Dai). (**C**) Self-assembly of MPP NPs and the ROS generation process. The positively charged surface of MPO can adsorb negatively charged polyphenol derivatives by electrostatic interactions. The Fe^3+^ can crosslink all the polyphenol derivatives together to form MPP NPs. Reprinted with permission from Ref. [74] (copyright 2018, Dai).

Natural polyphenols present potent antitumor activity against various cancer cell lines through multiple mechanisms. Still, inevitably, their low physiological stability and poor bioavailability can severely limit their biomedical applications [62]. Many drugs for tumor treatment are highly toxic to the kidneys and nerves, and they leak easily in the blood circulation, so the search for an MPN that can enhance the blood circulation of drugs has become a hot topic of interest for scientists. Polyethylene glycol is a synthetic polymeric material that can form polyphenolic derivatives, by aggregation with various phenolic compounds, because of its good unit-binding properties. Various reactions can anchor phenolic molecules to synthetic or natural polymers [71]. Several different PEG-polyphenols have been prepared by scholars based on these properties. Several papers that have reported various PEG-polyphenol derivatives, such as PEG-polyphenols formed with dopamine, catechol, 3,4-dihydroxyphenylalanine (DOPA), and 3,4-dihydroxycinnamic acid (DHCA), have gradually entered the limelight. The nanosystem created by PEG-polyphenols on the drug has good biocompatibility and acid-base stability in an aqueous solution, which can prolong the blood circulation in vivo and enhance the tumor accumulation effect, and PEG-polyphenols immobilized on the surface of the material can also reduce the non-specific adsorption of biomolecules in blood [72,73]. The PEG-polyphenol materials have similar properties to the MPNs. Therefore, from this perspective, Ju et al. designed a novel MPN capsule, in a simple and fast way, using dopamine coupled to hyaluronic acid (HA) and PEG in coordination with Fe^3+^, in which PEG-polyphenols have prolonged circulation time for adjustable cancer cell targeting (Figure 5A). The infiltration of PEG-polyphenols with HA both enhanced the binding effect of the capsule and reduced the non-specific interaction of the capsule with cancer cells [37].

### 2.8. Fe with Pt Prodrug-Polyphenol

Pt prodrug polyphenols are emerging artificial polyphenolic materials, which are high-quality polyphenols formed by combining chemotherapeutic prodrugs of platinum and phenolic compounds. Exhibiting both high-quality anticancer properties and good chelation with metal ions, Dai et al. designed an MPN consisting of Pt prodrug polyphenol and PEG-polyphenol by cross-linking with Fe^3+^ through self-assembly over two years (Figure 5B). This approach improved the tumor therapeutic efficacy of platinum prodrugs, but it also reduced the adverse side effects of platinum prodrugs with a loading efficiency of up to 23%. It successfully brought Pt prodrug-polyphenols into the limelight as materials for MPNs (Figure 5C). It has optimized previous MPNs and substantially improved the sequestration capacity of MPNs for drugs, opening up a new pathway for the design of MPNs [38,74].

### 2.9. Fe with Gossypol and PEG-Ce6-Polyphenol

**Figure 6:**
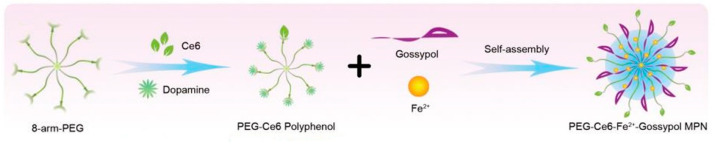
Schematic illustrations of the fabrication process of PFGs. Reprinted with permission from Ref. [39] (copyright 2021, Zhang).

Polyphenol ligands in MPN can substantially improve the effect of drugs on tumor treatment. Still, MPN formed by a single polyphenol ligand with Fe is more monotonous in improving treatment, and it is more challenging to kill tumor cells from the root cause [62]. From the perspective of tumor metastasis or recurrence and a therapeutic strategy to simultaneously target the primary tumor, Zhang et al. designed novel MPNs (PEG-Ce6-Fe^2+^-gossypol MPNs, PFGs) to evoke an efficient tumor immune response. The MPNs were designed with a polyphenol derivative (PEG-Ce6) formed from gossypol and PEG–chlorin e6 (Ce6) as a substrate, while they were ligated with Fe^2+^ [39] (Figure 6). Since gossypol exhibits certain anticancer properties and the similar structure of polyphenols makes it easy for natural drugs to be inserted into the MPN backbone stably [75], the MPNs have both good anticancer properties and biocompatibility. This is a typical MPN formed by hybrid polyphenols, which profoundly impacts the treatment of tumors.

## 3. Fe-Based MPNs in CDT

Chemodynamic therapy (CDT) is considered a promising modality for selective cancer therapy that leads to tumor cell death through in situ ROS toxicity, and it is an effective treatment for killing tumor cells by their endogenous catalytic hydrogen peroxide (H_2_O_2_), through the Fenton reaction or a Fenton-like reaction, to generate highly cytotoxic hydroxyl radicals (•OH). •OH is a ROS, which causes lipid peroxides (LPO) that destroy cell structure and integrity [29,30]. Its therapeutic efficiency is not limited by the light source and oxygen content, a current research hotspot, or a new strategy for tumor treatment [3,76]. Due to the strong TME selectivity and specificity, no external energy intervention, and excellent therapeutic efficiency, CDT can use endogenous chemical energy alone to induce protein inactivation, DNA damage, and LPO content and, finally, kill tumor cells endogenously and effectively to inhibit tumor cell growth, which has become a “green” clinical tumor treatment with great potential [76,77,78]. More importantly, this procedure greatly avoids the non-specific side effects (toxicity to normal tissues) and low efficiency (limited light penetration and oxygen dependence) associated with conventional cancer treatments such as chemotherapy and photodynamic therapy [79,80]. With the rapid development of metal–phenolic networks, the coating materials have been widely used in CDT for tumor treatment. Here, we will introduce the existing representative materials of Fe-based MPNs in CDT.

### 3.1. Fe with TA

TA-Fe^3+^ is the most common Fe-based MPN material, and it is often used as a drug coating, a catalyst for the Fenton reaction, and a pH-responsive agent [81]. During drug delivery, the MPN structure is well maintained, and when it reaches the acidic microenvironment of the tumor site, its MPN structure decomposes, releasing TA and Fe^3+^. Due to the good reducing property of TA, the Fe^3+^ released by the reaction can be chemically reduced to Fe^2+^. Compared with Fe^3+^, Fe^2+^ has higher catalytic activity. It thus reacts with H_2_O_2_ in the system to generate highly toxic •OH in TME, which substantially improves the efficiency of the Fenton reaction through a continuous supply of Fe^2+^. It forms a synergistic treatment with the released anticancer drugs and enhances the effect of CDT in killing tumor cells [63]. This shows that TA-Fe^3+^ can serve as an efficient source of iron ions for CDT. Interestingly, the antitumor mechanisms in which TA-Fe^3+^ wraps around different drugs may also somewhat show differences. Moreover, MPNs based on TA and Fe^3+^ have easy access to a wide range of adhesive materials from different templates due to their strong adhesion capabilities, such as polymeric micelles, nanosheets, hollow vesicles, nanotubes, and solid spheres [63].

**Figure 7:**
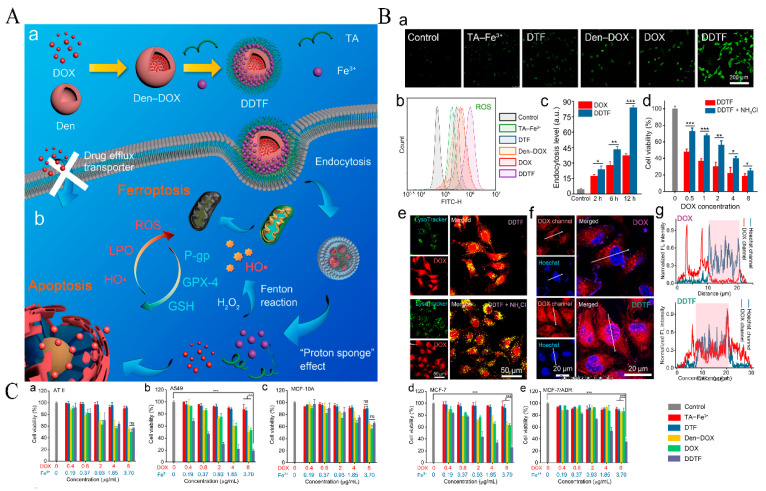
(**A**) Schematic showing the construction of DDTF nanocomplexes and their application for apoptosis/ferroptosis-mediated cancer therapy. (**a**) The dendrimer (Den)−DOX nanocomplex (Den−DOX) is first prepared by mixing DOX with the ethylenediamine core amine-terminated G4 polyamidoamine (PAMAM)dendrimer, and then tannic acid and iron ions are added to the above mixture to form MPNs (TA−Fe^3+^) on the surface of the Den−DOX nanocomplex, resulting in the formation of Den−DOX−Fe^3+^−TA (abbreviated as DDTF) nanodrug. (**b**) The significantly enhanced DOX uptake in the form of DDTF results in cell apoptosis via the induction of ROS generation. Meanwhile, the ferric ions in DDTF can facilitate the Fenton reaction and raise the ROS level. The elevated ROS level may inhibit the GPX-4 activity, block the synthesis of GSH, and finally hinder the clearance of intracellular LPO, which may eventually lead to cell ferroptosis. (**B**) ROS levels (reflected by the DCF fluorescence) of the A549 cells after various treatments, as observed by confocal microscopy (**a**) and measured by flow cytometry (**b**). (**c**) Endocytosis levels of DOX and DDTF (evaluated via the DOX fluorescence intensities, as measured by flow cytometry) in the A549 cells incubated with DOX or DDTF for various periods. * *p* < 0.05, ** *p* < 0.01, *** *p* < 0.001. (**d**) Viabilities of the DDTF-treated A549 cells without or with the pretreatment with NH_4_Cl (30 mM) at different DDTF concentrations (expressed as DOX concentrations). * *p* < 0.05, ** *p* < 0.01, *** *p* < 0.001. For (**a**–**d**), the cells without drug treatment were used as the control groups. (**e**) Confocal microscopic images of the DDTF-treated A549 cells, without or with the pretreatment with NH_4_Cl (30 mM), for 8 h. The lysosomes were stained with LysoTracker Green (abbreviated as LysoTracker). (**f**) Confocal microscopic images of the DOX or DDTF-treated A549 cells for 4 h. The nuclei were visualized by Hoechst 33342 (abbreviated as Hoechst). (**g**) Normalized line-scan fluorescence (FL) intensities of the white arrow marked positions in (**f**). The pink areas indicate the nuclear positions. The DOX content in (**a**–**c**) and (**e**–**g**) was 4 μg/mL. (**C**) Relative viabilities of the AT II (**a**), A549 (**b**), MCF-10A (**c**), MCF-7 (**d**), and MCF-7/ADR (**e**) cells subjected to the treatment with various concentrations of TA-Fe^3+^, DTF, Den-DOX, DOX, or DDTF. ** *p* < 0.01, *** *p* < 0.001, ns: nonsignificant difference. Reprinted with permission from Ref. [40] (copyright 2019, Guo).

In previous studies, apoptosis was considered the only programmed cell death pathway [82]. However, under a series of proofs, it has been found that the cell death mechanism of ferroptosis is different from apoptosis. It is a highly efficient at killing cancer cells [83,84]. It is an effective way to induce cancer cell death by generating high levels of intracellular reactive oxygen species through the Fenton reaction, which leads to oxidative damage of cellular components [85,86,87]. Adriamycin (DOX) is an anticancer chemotherapeutic drug that induces apoptosis. Still, inevitably, the low cellular uptake and inability to enter the nucleus of DOX are highly toxic to normal cells, which dramatically limits its performance in anticancer treatment [88]. Inspired by this, Guo et al. designed a nanocomposite drug (Den-DOX-TA-Fe^3+^, DDTF) composed of an MPN (TA-Fe^3+^) coating wrapped around a dendrimer (Den) and loaded DOX. The tumor treatment efficiency of CDT was improved by helping DOX accumulate neat toxicity in the nucleus and triggering cellular ferroptosis by co-producing ROS with the Fenton reaction (Figure 7A). By studying the data results of this nanocomposite drug DDTF, for triggering cellular ferroptosis, intracellular distribution, anticancer efficiency, the selective cancer cell killing effect, and biosafety assessment (Figure 7B), it was found that both ROS and LPO levels were significantly increased in treated cells, which indicated that DOX in DDTF could smoothly induce apoptosis. The TA-Fe^3+^ coating in it can effectively enhance the drug’s ability to generate cellular ferroptosis with good intracellular distribution, showing outstanding anticancer efficiency and specific toxic effects on cancer cells [40]. It combines the response characteristics of tumor cells treated with CDT and achieves a “win-win” effect.

**Figure 8:**
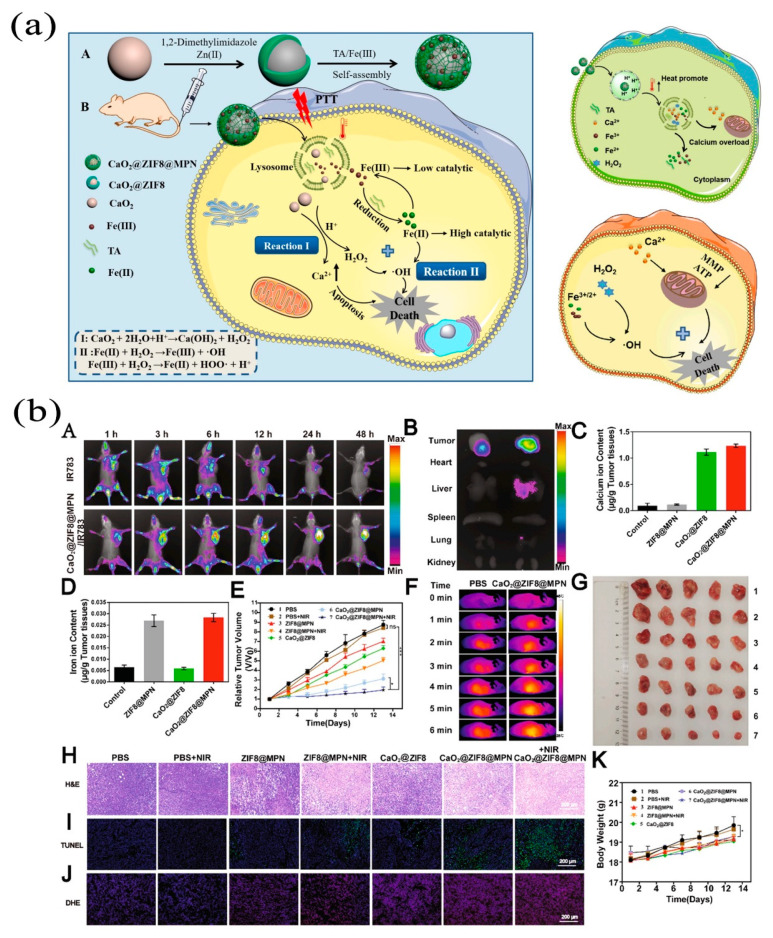
(**a**) Schematic illustration of CaO_2_@ZIF8@MPN as a pH-responsive free radical and ion nanogenerator for enhanced tumor CDT and ion therapy. (**A**) Preparation of CaO_2_@ZIF8@MPN and the (**B**) process of CaO_2_@ZIF8@MPN responds to lysosomal acid environments, and NIR laser irradiation promotes the release of Ca^2+^, H_2_O_2_, Fe^3+^, and TA, finally achieving H_2_O_2_ self-supply and iron ion self-circulation mediated enhanced CDT. The schematic illustration of CaO_2_@ZIF8@MPN could be internalized into the MCF-7 cells, generating sufficient H_2_O_2_, Fe^3+^, and Ca^2+^ via self-acidolysis. More importantly, the generated Ca^2+^ could be specifically distributed in mitochondria. Schematic illustration of explosive production of •OH, mitochondrial membrane potential, and ATP level decline, thus leading to the apoptosis of MCF-7 cells. (**b**) Anti-tumor effect in vivo. (**A**) Fluorescence images of tumor-bearing mice at different times after injection with free IR783 and CaO_2_@ZIF8@MPN/IR783; (**B**) the fluorescence images of ex vivo tumors and major organs after injection with free IR783 and CaO_2_@ZIF8@MPN/IR783 for 48 h; (**C**,**D**) the Ca^2+^ and Fe^3+^ content in tumors after being treated with different formulations (n = 3); (**E**,**K**) relative tumor volume and body weight of tumor-bearing mice after treatment (n = 5); (**F**) thermal imaging of tumor-bearing mice after injected with CaO_2_@ZIF8@MPN and then irradiated by NIR laser for different times (808 nm, 2 W/cm^2^); (**G**) photos of tumor tissues after treated for 14 d (n = 5); (**H**,**I**) H&E staining and TUNEL staining of tumor tissues collected from different groups, scale bar: 200 μm; (**J**) ROS generation in tumors from mice receiving different treatments, scale bar: 200 μm. * *p* < 0.05 and *** *p* < 0.001 determined by Student’s *t*-test. Reprinted with permission from Ref. [41] (copyright 2021, Liu).

The Fenton reaction of Fe^2+^ will be oxidized to Fe^3+^ by H_2_O_2_, so it will severely limit the efficiency of •OH production [89,90]. Although the strategies of direct delivery of H_2_O_2_ or the use of glucose oxidase (GOx) to catalyze the generation of H_2_O_2_ can significantly improve the efficiency of the Fenton reaction [91], there will still be inevitable leakage of the substance in the circulation and limitation by the oxygen content of the tumor. Therefore, with the help of the properties of MPN, it can help effective drug delivery. Liu et al. chose TA-Fe^3+^ and designed a nanoparticle (CaO_2_@ZIF8@MPN) made of ZIF-8 piggybacked calcium peroxide (CaO_2_) that can enhance CDT using the properties of pH-responsive radicals [41] (Figure 8a). CaO_2_, as a metal peroxide with a peroxide ion (O_2_^2-^), decomposes under dilute acidic conditions to form Ca(OH)_2_ and H_2_O_2_ with modifiable Fenton chemistry to serve as an alternative source of H_2_O_2_ in tumor treatment [92,93]. ZIF-8 is a typical metal-organic framework material that has attracted much attention in the biomedical community in recent years. Its structure can encapsulate specific molecular particles, often studied as drug delivery carriers [94,95,96]. The nanoparticles cleverly exploit the pH responsiveness of TA-Fe^3+^ in TME, releasing Fe^2+^ to increase the concentration of reactants to enhance the efficiency of the Fenton reaction and initiate the Fenton reaction within the tumor without external energy input [77]. An advantage of CaO_2_@ZIF8@MPN is that it does not contain chemotherapeutic drugs, and its rapid degradation in vivo can effectively avoid the accumulation of inert carriers in vivo and avoid the toxicity triggered [41]. This method integrates the strategies of H_2_O_2_ self-supply, Fe^3+^ self-circulation, and intracellular Ca^2+^ overload properties to achieve synergistic treatment of tumors with drug-free CDT and ions, which is an innovative point that further increases the prospect of therapeutic approaches of CDT for tumors.

**Figure 9:**
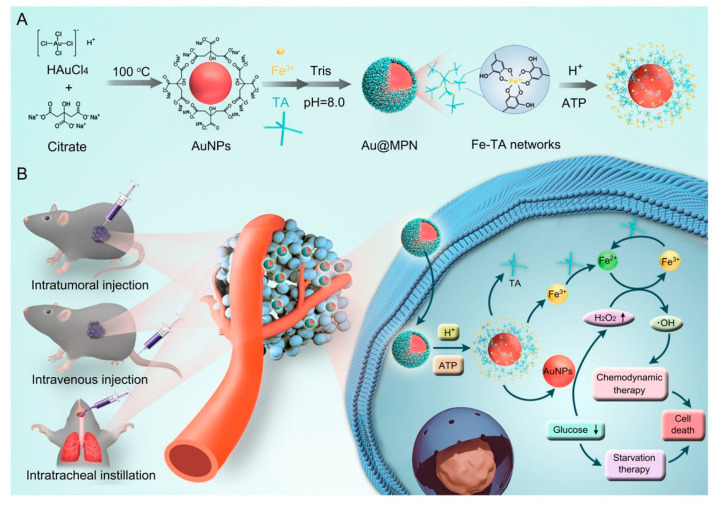
Schematic illustrations of the Au@MPN with the ability to self-supply H_2_O_2_ for enhanced chemodynamic therapy. (**A**) Preparation of Au@MPN and their disassembly process in the presence of ATP or in an acidic environment. (**B**) The antitumor mechanism of Au@MPN for enhanced chemodynamic therapy. Reprinted with permission from Ref. [42] (copyright 2022, Peng).

**Figure 10:**
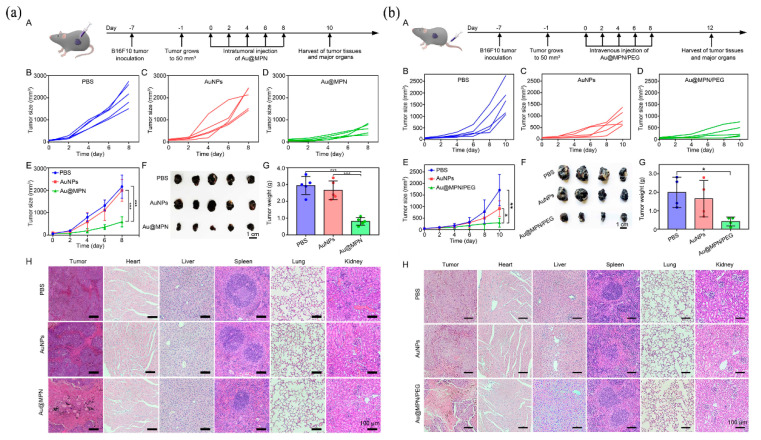
(**a**) Antitumor effect of Au@MPN after intratumoral injection. (**A**) Schematic illustration of the antitumor process of Au@MPN intratumoral injection. (**B**–**D**) Individual tumor growth curves from mice treated with PBS, AuNPs, and Au@MPN. (**E**) Relative tumor volumes from mice after the indicated treatments. (**F**) Photos of tumors extracted from mice receiving the indicated treatments. (**G**) The weights of tumor tissues from mice receiving the indicated treatments. (**H**) Representative H&E staining images of tumor tissues and major organs collected from mice with the indicated treatments. (**b**) Antitumor effect of Au@MPN after intravenous injection. (**A**) Schematic illustration of the antitumor process of Au@MPN/PEG intravenous injection. (**B**–**D**) Individual tumor growth curves from mice treated with PBS, AuNPs, and Au@MPN/PEG. (**E**) Relative tumor volume from mice receiving the indicated treatments. (**F**) Photos of tumors extracted from mice receiving the indicated treatments. (**G**) The weights of tumor tissues from mice receiving the indicated treatments (**H**) Representative H&E staining images of tumor tissues and major organs collected from mice receiving the indicated treatments. * *p* < 0.05, ** *p* < 0.01, and *** *p* < 0.005. Reprinted with permission from Ref. [42] (copyright 2022, Peng).

Glucose oxidase has been reported for the self-supply of H_2_O_2_ for CDT [97] and the oxidation of intracellular glucose to produce H_2_O_2_ [98]. Peng et al. selected a facile method to construct a core-shell nanoplatform (Au@MPN), with ATP and low pH dual response disassembly, by coating gold nanoparticles (AuNPs) with TA-Fe^3+^ coating [42] that enables self-supply of H_2_O_2_ for autocatalytic Fenton reaction as a therapeutic system to enhance CDT efficiency (Figure 9). Due to their ability to successfully combat cancer cells and cellular non-toxicity, as well as their natural enzyme-like activity [99], AuNPs exhibit excellent therapeutic efficacy under the MPN coating. It was evident from the in vivo antitumor assessment test that mice injected with Au@MPN exhibited considerably stronger tumor suppression than those injected with PBS and AuNPs, while the photographs and weights of the extracted tumors were consistent with the results of the relative tumor volumes. Furthermore, H&E staining showed cytoarchitectural damage and sparse nuclei in tumor tissues of the Au@MPN group, which strongly demonstrated the effective antitumor activity of Au@MPN. Not surprisingly, the environment of ATP and low pH-induced Au@MPN to undergo double-response decomposition, releasing Fe^3+^ and further converting to Fe^2+^ by TA as a highly catalytically efficient Fenton agent, which could effectively inhibit the growth of primary tumors and metastatic melanoma lung tumors (Figure 10). Meanwhile, the AuNPs exposed after the decomposition of the MPN coating catalyze the in situ generation of H_2_O_2_ from glucose oxidation, which further enhances •OH production by reacting with Fe^2+^ [98]. This is another new achievement of TA-Fe^3+^ as an MPN material for the self-supply of H_2_O_2_ and enhanced CDT by autocatalytic Fenton reaction in tumor treatment.

**Figure 11:**
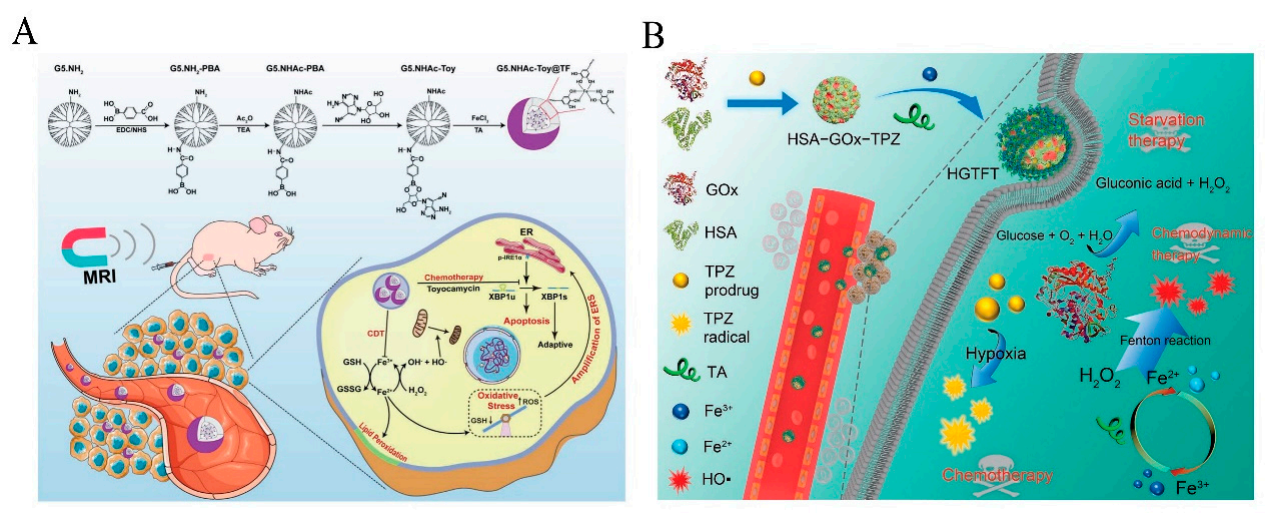
(**A**) Schematic illustration of the preparation of G5.NHAc-Toy@TF nanocomplexes for MR imaging and chemotherapy/CDT of tumors in vivo through ERS amplification. Reprinted with permission from Ref. [43] (copyright 2022, Wang). (**B**) Schematic diagram showing the fabrication of HGTFT nanoreactors and their applications for starvation therapy, CDT, and chemotherapy. Reprinted with permission from Ref. [44] (copyright 2020, Guo).

CDT can be used both as a stand-alone treatment and to form a synergistic treatment with chemotherapy. The general approach is to use a material coating to wrap the chemotherapeutic drug to reduce the toxicity caused by the drug in transit and to improve the therapeutic effect on tumor cells [54]. A representative study of chemotherapy/CDT synergistic therapy is that of the nanoparticles (G5.NHAc-Toy@TF) designed by Wang et al., which consists of a TA-Fe^3+^ coating wrapped with endoplasmic reticulum stress (ERS) of toyocamycin (Toy) anticancer drugs for enhanced combination antitumor treatment (Figure 11A). Since MPN dissociates and releases TA, it can reduce Fe^3+^ to Fe^2+^, thus significantly promoting the Fenton response. Tumor cells adopt aerobic glycolysis as the central metabolic pathway, producing lactic acid, leading to an acidic tumor environment that facilitates efficient CDT production of ROS by the G5.NHAc-Toy@TF complex. Experiments have confirmed that this nanocomplex upregulates ROS and downregulates glutathione (GSH) and GPX-4, allowing LPO to accumulate in the cells and leading to cellular ferroptosis [43].

**Figure 12:**
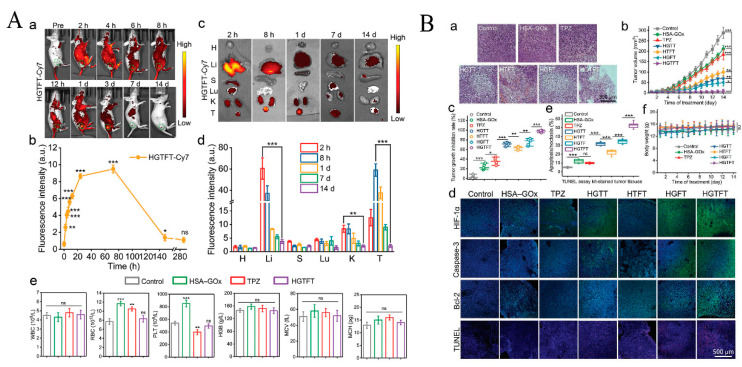
(**A**) (**a**) Time-dependent fluorescence pictures of the 4T1 tumor-bearing mice that were i.v. injected with HGTFT-Cy7 and (**b**) corresponding fluorescence levels of HGTFT-Cy7 in the tumor areas. “Pre” indicates the mouse before drug treatment. The tumor regions of the mice in (**a**) were marked using green dotted circles. * *p* < 0.05, ** *p* < 0.01, and *** *p* < 0.001, versus control, ns: nonsignificant difference. (**c**) Ex vivo fluorescence pictures of the major organs and tumor regions of the HGTFT-Cy7-injected mice sacrificed at various time points and (**d**) corresponding fluorescence levels. ** *p* < 0.01 and *** *p* < 0.001. The pictures in (**a**,**c**) and the intensities in (**b**,**d**) are based on the fluorescence of Cy7. The abbreviations H, Li, S, Lu, K, and T in (**c**,**d**) represent the heart, liver, spleen, lung, kidneys, and tumor, respectively. (**e**) Routine blood analysis data of the mice sacrificed on the 14th day after the intravenous injection of PBS (control), HAS-GOx, TPZ, or HGTFT. ** *p* < 0.01 and *** *p* < 0.001, versus control, ns: nonsignificant difference. The blood indexes WBC, RBC, PLT, HGB, MCV, and MCH represent numbers of white blood cells, red blood cells, platelets, hemoglobin concentration, mean corpuscular volume, and mean corpuscular hemoglobin, respectively. (**B**) (**a**) Representative optical microscopic pictures of the H&E-stained tumor slices from the 4T1 tumor-bearing nude mice, taken at day 14, after being treated with different formulations. (**b**) Tumor growth profiles of the mice in the different groups. * *p* < 0.05, ** *p* < 0.01, and *** *p* < 0.001. (**c**) Tumor growth inhibition rates of different formulations. * *p* < 0.05, ** *p* < 0.01, and *** *p* < 0.001. (**d**) Representative immunofluorescence staining results of HIF-1α, caspase-3, and Bcl-2, as well as representative TUNEL assay results of tumor tissue slices from the 4T1 tumor-bearing nude mice, sacrificed at day 14, after being treated with different formulations. (**e**) Apoptosis/necrosis rates determined by the TUNEL assay. *** *p* < 0.001, ns: nonsignificant difference. (**f**) Body weight changes of the mice subjected to the treatments of different formulations. “ns” represents a nonsignificant difference. Reprinted with permission from Ref. [44] (copyright 2020, Guo).

The synergistic treatment strategies of chemotherapeutic drugs and proteins are also explored, except for the synergistic treatment of therapies. For example, after combining tilazamine (TPZ) and human serum albumin (HAS), Guo et al. designed a nanoparticle (HSA-GOx-TPZ-Fe^3+^-TA, HGTFT), with the help of GOx and TA-Fe^3+^ coating, to achieve a drug/protein combination and chemotherapy/CDT synergy for a better therapeutic effect of CDT [44] (Figure 11B). TPZ is a bioreductant and anticancer agent with selective cytotoxicity against hypoxic cells in solid tumors [94,100], so HAS is a water-soluble plasma protein with good biocompatibility, physiological stability, and good tumor targeting. It is now an excellent drug carrier for tumor treatment [101]. In HGTFT, the presence of the prodrug TPZ can be converted to the highly toxic TPZ radical, under hypoxic conditions, for hypoxia-induced chemotherapy [102]. Meanwhile, GOx generates exogenous H_2_O_2_ for the Fenton reaction, starvation therapy consumes glucose, and hydroxylated TPZ radical-mediated chemotherapy consumes O_2_ (Figure 12). However, the nanoparticles contain only Fe^3+^, which does not contain the Fe^2+^ required for the Fenton reaction, whereby TA acts as a reducing agent to promote Fe^2+^ in an acidic environment (e.g., endogenous/lysosomal) by accelerating the conversion of Fe^3+^/Fe^2+^ generation for efficient and sustainable CDT and metal ion interference therapy [44]. The synergistic effects of starvation therapy, chemotherapy, and CDT can significantly damage tumor tissues. The importance of this Fe-based MPN coating to provide sustainable CDT is further confirmed compared to the data from controls without Fe^3+^ or TA.

### 3.2. Fe with EGCG

EGCG has strong iron chelating activity, stable coordination with Fe^3+^ and Fe^2+^, the strong free radical scavenging ability after forming MPN, and the ability to protect cells from LPO. In contrast, EGCG can effectively and specifically inhibit the tumor proteasome activity associated with the induction of apoptosis in cancer cells. Still, because it can bind biomolecules non-specifically and accumulate rapidly in major body organs, it is detrimental to blood circulation and tumor-specific delivery [103]. As a result, EGCG is generally not used alone. MPNs formed by EGCG and iron ions are often encapsulated as nanoparticles with chemotherapeutic drugs to enhance tumor treatment, or they are used with other composites to form MPN nanocoatings to encapsulate drug molecules.

In the Fenton reaction, the highly reactive Fe^2+^ is easily converted to the less reactive Fe^3+^, which is the main reason for limiting the therapeutic efficiency of CDT [104]. It has been reported that the efficiency of the Fenton reaction of CDT can be improved at higher relative temperatures to increase the yield of •OH. Therefore, photothermal agents are often introduced to convert near-infrared (NIR) light into PTT to enhance tumor suppression by CDT [105]. Inspired by this, Yu et al. designed novel self-assembled metal–polyphenolic nanodrugs (FeEP-NPs) using MPNs composed of Fe^2+^ and EGCG with PVP as a stabilizer to further improve the therapeutic efficiency of CDT and some nanomaterials with complex synthesis methods, poor reproducibility, low stability, and high cost. This nanodrug is simple in composition, versatile in function, and has a unique nanoplatform to achieve enhanced CDT-effective tumor elimination via mild PTT [105]. FeEP-NPs can act as catalysts for the Fenton reaction to remove tumor cells in a mildly acidic environment selectively.

Interestingly, when H_2_O_2_ was added to trigger the Fenton reaction, free Fe^2+^ in the solution was rapidly converted to Fe^3+^ in contrast to the large amount of Fe^2+^ still present in the solution containing EGCG. This suggests that EGCG released from MPN can significantly improve the efficiency of the Fenton reaction by continuously supplying Fe^2+^, thus obtaining an efficient CDT effect. In particular, its effect was considerable in an in vivo therapeutic trial of mild PTT-enhanced CDT, and the tumor suppression rate exhibited was particularly outstanding. The higher antitumor efficiency of FeEP-NPs, based on synergistic therapy, was further illustrated by tests such as the amount of apoptosis on the degree of tumor tissue damage by FeEP-NPs [50]. The ability of Fenton nanotherapeutic FeEP-NPs, containing EGCG and Fe^2+^ with high efficiency, safety, and low side effects, as well as their mild PTT-enhanced CDT with PA imaging-guided tumor suppression strategy, provides a broad application prospect for future clinical treatment of tumors.

### 3.3. Fe with PEG-Polyphenol

**Figure 13:**
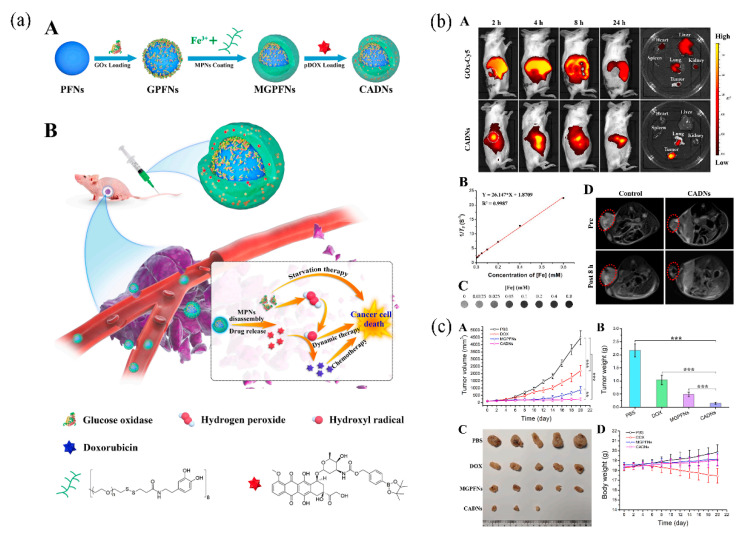
(**a**) Schematic illustrations for (**A**) the preparation of CADNs and (**B**) their application in cancer combination therapy. (**b**) (**A**) In vivo fluorescence imaging of 4 T1 tumor-bearing mice at different times (2, 4, 8, and 24 h) after being intravenously injected with GOx-Cy5 or GOx-Cy5 loaded CADNs. Main organs and tumors were collected after 24 h. White dot circles represented tumor sites. (**B**) Transverse relaxation values (1/T2) of CADNs varied with various Fe concentrations. (**C**) T2-weighted MRI images of CADNs with various Fe concentrations measured at 3.0 T. (**D**) In vivo T2-weighted MRI images of four T1 tumor-bearing mice before and after being injected with PBS or CADNs. Red dot circles indicated tumors. (**c**) Evaluation of in vivo antitumor activities of PBS, DOX, MGPFNs, and CADNs in MCF-7 cells bearing nude BALB/c mice. (**A**) Records of tumor volumes at different set time points in the treatment process. ** *p* < 0.01 and *** *p* < 0.001. (**B**) Weights and (**C**) photographs of excised tumors at the end of treatment. *** *p* < 0.001. (**D**) Body weight changes of nude mice in various groups during the therapeutic process. Reprinted with permission from Ref. [55] (copyright 2022, Chen).

PEG-polyphenols can form MPNs to cooperate with other therapeutic agents based on metal–polyphenolic coordination. Commonly, MPNs formed by Fe^3+^ and PEG-polyphenols are often used as hollow capsules to deliver chemotherapeutic drugs, prolong the circulation of medicines in the blood, and enhance the targeted therapy of tumors [106]. Considering the high level of ROS in tumor cells compared to normal cells and the problem of nutrient supply to tumor cells, Chen et al. decided to use starvation therapy and the metal ion-mediated Fenton reaction of CDT in synergy with chemotherapeutic drugs. They designed comprehensive nanocarriers (CADNs) in which, mixed with nanoparticles Fe_3_O_4_, GOx, and prodrug of DOX (pDOX), Fe^3+^–PEG-polyphenol was employed as the coating of MPNs [55] (Figure 13). GOx can catalyze the rapid conversion of glucose to gluconic acid, thus depriving tumor cells of glucose and cutting off the nutrient supply to reach the effect of starvation therapy. Meanwhile, H_2_O_2_, as a by-product of the enzymatic reaction, can be decomposed by the Fenton reaction, mediated by Fe^3+^/Fe^2+^, to produce the highly toxic •OH, which can achieve the effect of CDT and kill the tumor cells with chemotherapeutic drugs. Notably, under the pH responsiveness of the MPNs, the nanocarriers can rapidly release drugs in acidic or reducing environments and convert pDOX to DOX under oxidative stress. The integrated drug delivery nanocarriers of GOx and pre-delivery can promote the Fenton reaction and produce significant combined therapeutic effects. CADNs kill tumor cells by the ferroptosis and chemotherapy, via DOX, of tumor cells [55]. This cascade amplification delivery system provides an effective therapeutic platform for selective tumor growth inhibition and the suppression of multidrug resistance (MDR) in cancer.

### 3.4. Fe with Hybrid Polyphenol

**Figure 14:**
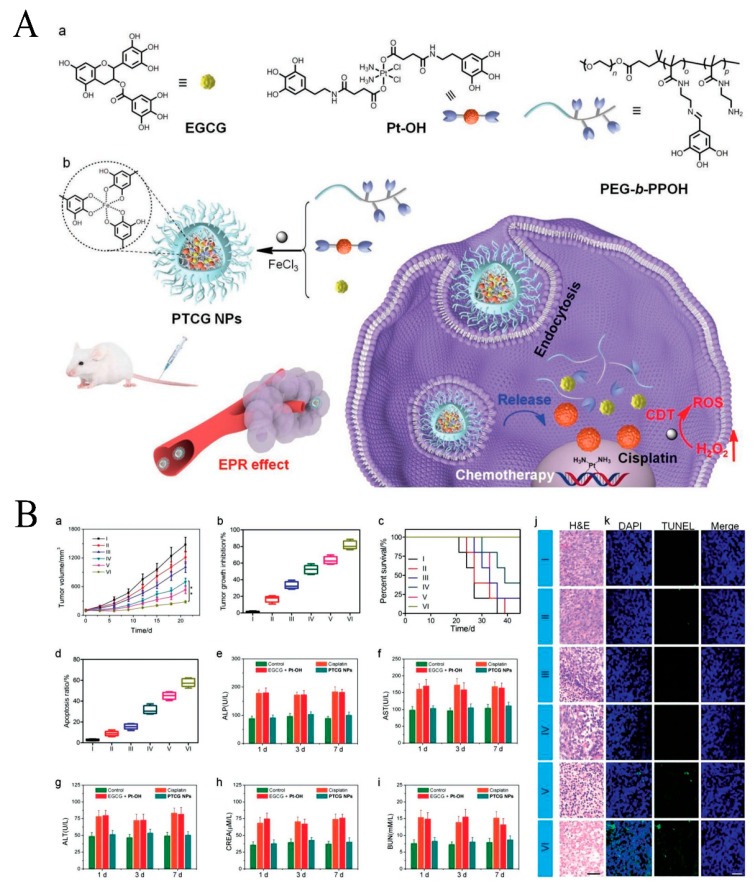
(**A**) (**a**) The chemical structures and cartoon illustration of the building blocks (EGCG, Pt-OH, PEG-b-PPOH) used for the preparation of PTCG NPs. (**b**) High tumor accumulation of PTCG NPs through the EPR effect followed by cellular internalization. The nanomedicine opens a floodgate, specifically in cancer cells, to release drugs (EGCG and cisplatin) and ROS to realize chemo/chemodynamic therapy. (**B**) (**a**) Tumor growth–inhibition curves and (**b**) tumor growth–inhibition rate of the mice bearing HepG2 tumors after different treatments (n = 5), ** *p* < 0.01. (**c**) Survival rate of the mice after different treatments. (**d**) Apoptosis ratio of the tumor cells for the mice after different treatments. (**e**–**i**) Blood biochemistry tests of ALP (**e**), AST (**f**), ALT (**g**), CREA (**h**), and BUN (**i**) from the mice treated with different formulations. Untreated healthy mice were used as the control. (**j**) H&E and (**k**) TUNEL staining of the tumor tissues from the mice treated with different formulations. The scale bar = 100 µm. I, Control; II, EGCG NPs; III, EGCG + Pt-OH; IV, cisplatin; V, Pt-OH NPs; VI, PTCG NPs. Data are expressed as means ± s.d. Reprinted with permission from Ref. [54] (copyright 2019, Ren).

Cisplatin is an ideal therapeutic partner for CDT because of its broad anticancer spectrum, effectiveness in depleted cells, and strong action [107]. It enables chemotherapy/CDT for cancer treatment by self-supplying H_2_O_2_ for Fenton response. However, cisplatin has some toxicity when used for cancer treatment and is prone to side effects [108]. Pt prodrug-polyphenol is a novel type of polyphenol that has emerged in recent years, combining the anticancer properties of a platinum prodrug with the characteristics of a polyphenol. Accordingly, Ren et al. used a 5-hydroxydopamine-modified Pt prodrug-polyphenol (Pt-OH) in combination with EGCG. They modified PEG-polyphenol (PEG-b-PPOH) for the use of cisplatin in chemotherapy and the perspective of CDT. An organic therapeutic nanodrug (PTCG NPs) with high loading capacity was constructed under the coupling effect of Fe^3+^ [54]. This is the first Fe-based MPN incorporating three polyphenols (Figure 14).

Due to the cleavage of the imine linker and the activation of Pt-OH for cisplatin at low pH, the active drug is effectively released from polyethylene glycolic NPs after internalization by HepG2 cells, and the generated cisplatin raises H_2_O_2_ levels through an intracellular cascade reaction that is catalyzed by Fe^3+^ to produce toxic ROS, thereby enhancing the effectiveness of tumor treatment with the introduction of CDT. It is noteworthy that EGCG becomes an ideal chemical partner for platinum-based drugs during this treatment because the presence of EGCG significantly inhibits telomerase expression, which makes cancer cells more sensitive to chemotherapy [109]. Equally important, the encapsulation of Fe^3+^ with PEG-polyphenols’ structure prolongs the platinum prodrugs’ time in circulation and protects tissues and organs from neurotoxicity and nephrotoxicity [110]. In PTCG NPs, metal-polyphenolic complexes form the core and stabilize the assembly. At the same time, PEG chains dominate the outside world as a shell of NPs interacting with water, thus stabilizing the multicomponent PTCG NPs.

On the one hand, the decomposition of PTCG NPs will make the H_2_O_2_ diffuse and contact the catalyst more easily, which is more favorable for the Fenton reaction. On the other hand, under acidic conditions, the Fenton reaction will maintain a high response rate due to the formation of Fe^3+^. From the in vivo antitumor experimental comparison, it is evident that EGCG NPs exhibited less than ideal anticancer effects, indicating that the presence of EGCG is a facilitator of treatment. The anticancer effect of EGCG, alone, is far less potent than the drug’s. At the same time, the tumor growth of mice injected with PTCG NPs was greatly inhibited, with an inhibition rate of 81.6%, which convincingly confirms that the combination of chemotherapy and CDT can substantially enhance its antitumor performance. The results of this experiment were attributed to EPR effects, stimulation of responsive drug release, elevated ROS, synergistic antitumor effects, and chemotherapy/CDT by Ren et al. [54]. This approach has not only opened a new path for MPNs in CDT but also inspired a strategy for MPNs with hybrid polyphenols to improve tumor treatment efficacy.

## 4. Fe-Based MPNs in PTT

Phototherapy (PTT) has become a popular cancer treatment method in recent years, and it is used to treat many kinds of cancer. PTT is a method to stimulate phototherapeutic reagents to kill tumor cells by irradiating the lesion area with light sources, especially NIR light sources, to achieve the treatment purpose. Photodynamic therapy (PDT) and photothermal therapy (PTT) are the two primary forms of phototherapy. PDT is mainly activated by photosensitizers at specific wavelengths, and under oxygen conditions, energy is transferred to oxygen to generate ROS, which induces apoptosis or necrosis to remove tumor cells from the body [111,112,113]. PDT has been used in the clinical treatment of cancer in recent years because of its reproducibility, low toxic side effects, lack of inherent drug resistance, and non-invasiveness [6,114,115]. However, there are some problems with this therapy to be solved by the Prime Minister, which lacks targeting and cannot act precisely and minutely on the tumor site. The second is the choice of photosensitizer, which is not easily delivered to the tumor site in the body, and the effect of photodynamic therapy will be reduced when the tumor site is hypoxic [1,116]. PTT is a non-invasive therapy, which is performed by injecting materials with a photothermal conversion effect into the human body, making them gather at the tumor site, and irradiating the tumor site with exogenous light so that the light energy is converted into heat energy to warm up the local area. Thus, this therapy has the advantages of certain targeting and low damage to other tissues [5,8,117]. The therapy also has some shortcomings, such as low photothermal conversion efficiency, insufficient tumor accumulation and cellular uptake, and tumor metastasis, which affect the efficacy of PTT, i.e., it is worth exploring whether the development of new nanomaterials can successfully solve the above problems, and exploring how to improve the photothermal conversion efficiency is the key, which, in turn, enhances the efficacy of PTT.

Among them, nanomaterial-based phototherapeutic systems overcome many drawbacks of traditional small molecules and attract much attention. MPNs are nanomaterials composed of phenolic compounds and metal cation ligand pairs that can be self-assembled with various functions [7,118], among which Fe is more commonly used in MPNs, and Fe^3+^ can combine with multiple phenols to make MPNs have good biocompatibility and enhanced photothermal efficiency, which can be used as a good photothermal agent to enhance the PT effect. In addition, Fe-structured MPNs can also be used as photosensitizers to promote ROS generation and induce cancer cell death. MPNs can not only be combined with PT to build a diagnostic and therapeutic nanophototherapy system but they can also encapsulate chemotherapeutic drugs and various photosensitizers to build a more functional nanophototherapy system, which can play the role of protecting photosensitizers and photothermal agents, ensuring that multiple therapies play a synergistic role and using a combination of therapies, which have extensive prospects in clinical application.

### 4.1. Fe with TA

Since the strong coordination interactions between phenolic compounds (e.g., GA, EGCG) of the corresponding MPN and iron ions contribute to the formation of different charge transfer bands and the appearance of new absorption in the near-infrared region, making MPNs good photothermal nano-agents [14,119,120] as well as drug-carrying tools, Fe-TA has an excellent photothermal conversion rate and is used in various therapeutic systems. However, since PT, as a therapy, requires high levels of photothermal agents and photosensitizers, efficient photothermal agents are needed to enhance the efficacy of PT. Based on the above, Meng et al. developed a PID@Fe-TA drug delivery system using Fe^3+^-TA as DOX and indocyanine green (ICG) encapsulated in the outer layer of the PID complex formed by polyethyleneimine (PEI), which was used as a drug delivery vehicle by interpreting the inner layer of the drug under laser irradiation [45]. According to the experimental results, after increasing the concentration of PID@Fe-TA, the local temperature increased more under the same laser conditions of irradiation and reached the required temperature for PT. This result may be due to the photothermal effect of Fe^3+^-TA, which shows that Fe^3+^-TA has much more potential than just the photothermal effect. Additionally, the photothermal agent ICG in the modified delivery system dramatically reduces the loss due to the protective effects of Fe^3+^-TA and PEI. The local temperature increase under laser irradiation also drives the photothermal effect of Fe^3+^-TA. The choice of non-toxic materials and the simple synthesis route of this system provide new ideas to other teams.

**Figure 15:**
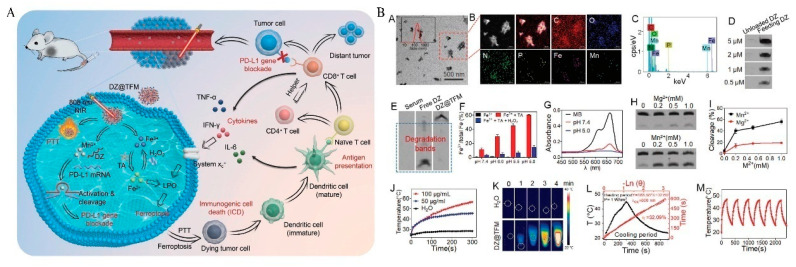
(**A**) Schematic illustration of DZ@TFM for PTT-enhanced cyclically amplified tumor ferroptosis-immunotherapy. (**B**) Preparation and characterizations of DZ@TFM. (A) TEM image and dynamic light scattering (inset), (B) elemental mapping images, and (C) energy disperse X-ray spectroscopy (EDS) analysis of DZ@TFM. PAGE images for (D) DZ loading capacity characterization and (E) protection effect against serum. (F) Measurement of Fe^2+^ content with different treatments (n = 3). (G) The degradation of MB under pH 5.0 or 7.4 conditions in the presence of H_2_O_2_. (H) PAGE images showing DZ activity for substrate cleavage and (I) the cleavage quantification (n = 3). (J) Photothermal curves of DZ@TFM with 808 nm laser irradiation at different concentrations. (K) Photothermal images of DZ@TFM solutions with laser exposure. (L) Calculation of the photothermal conversion efficiency at 808 nm. (M) Continuous irradiation–cooling cycle profiles of DZ@TFM. Reprinted with permission from Ref. [46] (copyright 2019, Liu).

**Figure 16:**
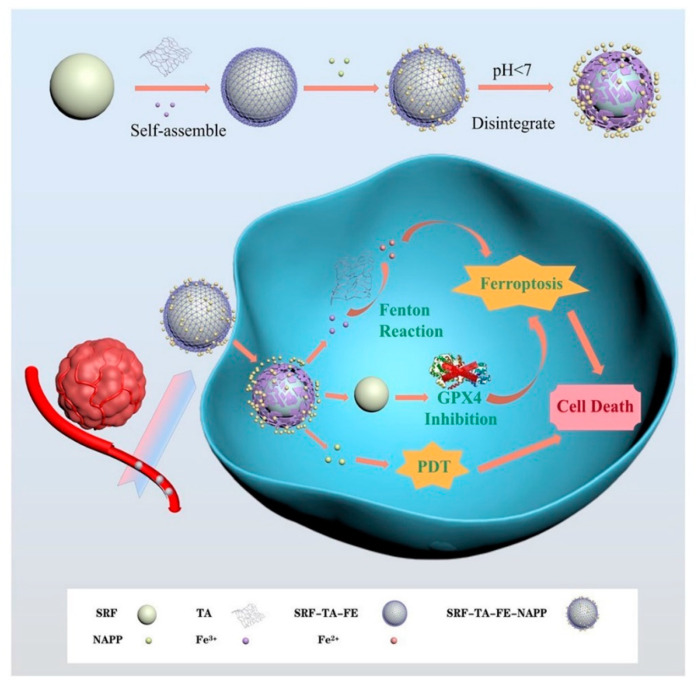
Preparation and mechanism diagram of SRF@Fe^III^TA–NAPP. Due to the low pH environment inside tumor cells, Fe^III^TA cleavage releases SRF to induce ferroptosis, NAPP is activated under a specific wavelength (660 nm) for PDT, Fe^3+^ is under the action of TA, and the oxidation–reduction into Fe^2+^ is used for the occurrence of the Fenton reaction, which catalyzes the high concentration of ROS in tumor cells to enhance ferroptosis and the efficacy of PDT. Reprinted with permission from Ref. [47] (copyright 2022, Zhou).

In the study by Liu et al., the DZ@TFM drug delivery system was also fabricated using Fe^3+^ and TA ligand chelation, further demonstrating the good photothermal conversion of Fe^3+^-TA [46]. Fe^3+^-TA effectively protects the drug from biodegradation, while the iron-structured MPNs have a reasonable photothermal conversion rate, enhancing PT’s effectiveness for tumor treatment [7]. The study observed concentration-dependent heating kinetics by calculating the photothermal conversion rate at 808 nm. It attributed the photothermal conversion ability to the Fe-TA structure [121] (Figure 15), which also favorably demonstrates the unlimited potential of Fe^3+^-TA in enhancing the efficacy of PTT and much more. In addition to its good photothermal conversion, Fe-TA has a good pH response and can act as a “protective layer” for the drug, greatly reducing the drug’s loss and increasing its efficacy. Efficient drug transport to tumor sites has been a hot topic of research by scientists, among which Zhou et al. designed a nanoplatform (SRF@Fe^III^TA–NAPP), combining MPN-based ferroptosis and PDT, to obtain better therapeutic effects through combined PDT and ROS treatment (Figure 16). Due to the good pH responsiveness of Fe^3+^-TA, the MPN structure under neutral conditions is stable.

In contrast, the MPN structure under acidic conditions is cleaved, which can successfully protect the drug from the normal pH value of the human body. In contrast, the cleaved release of photosensitizer NAPP in the tumor microenvironment promotes PDT. The released Fe^3+^ generates a large amount of ROS through the Fenton reaction to induce and promote cellular ferroptosis. The generation of ROS and induction of cell death is the principle of PDT. Thus, Fe^3+^-TA not only plays a protective role but it also indirectly promotes PDT [47].

Fe^3+^-TA can not only promote PT efficacy but it can also combine with CDT/chemotherapy for tumor treatment. When Fe^3+^-TA accumulates intracellularly, to a certain extent, Fe^3+^ in iron-structured MPNs is reduced to Fe^2+^ by TA under tumor cell microconditions. On the one hand, Fe^2+^ accumulation in tumor cells, to a certain amount, causes ferroptosis, an iron-dependent form of regulated cell death caused by unrestricted lipid peroxidation and subsequent membrane damage, which induces cell death [122]. On the other hand, reducing Fe^3+^ to Fe^2+^ promotes the production of •OH and enhances the Fenton reaction, which induces cancer cell death without harming normal cells and enhances CDT. In conclusion, Fe^3+^-TA is used in PT more as a photothermal agent, followed by coating on the drug and preparation in hollow capsules, nanoparticles, etc. Developing drug platforms with multimodal therapeutic tumors using Fe^3+^-TA has great promise and unlimited potential for clinical applications.

### 4.2. Fe with EGCG

EGCG has been studied as an excellent anticancer drug that can achieve excellent anticancer effects and reverse drug resistance when combined with DOX [123]. However, many investigations have shown that the in vivo application of EGCG is limited due to its instability, poor bioavailability, low adsorption, and rapid metabolism [124]. Therefore, many macromolecules, such as polyethylene glycol, keratin, bovine serum albumin, and chitosan, have been applied to improve the stability of teicoplanin and enhance its therapeutic effects [125,126,127,128]. Given the abundance of phenolic hydroxyl groups in EGCG, which not only gives them high antioxidant properties but also the ability to chelate with metals, they have the potential to act as organic ligands for metal–phenolic networks, which would greatly enhance their stability and tumor therapeutic efficacy.

**Figure 17:**
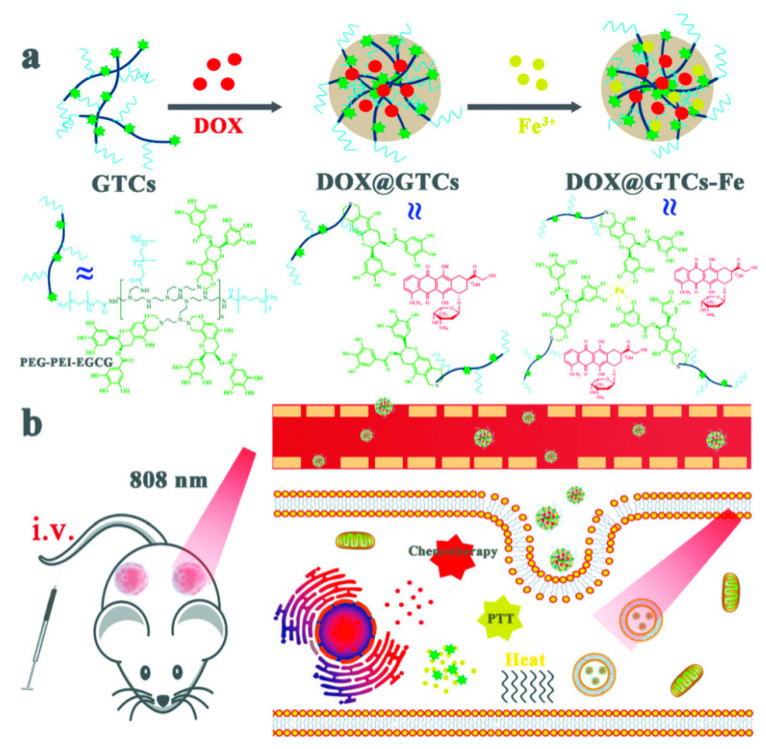
Schematic illustrations of (**a**) covalently-assembled GTCs from EGCG and PEI–PEG to load DOX for DOX@GTCs nanoparticles and, further, for DOX@GTCs–Fe by chelating with ferric ions and (**b**) intravenous injection of both nanoparticles for effective chemo-photothermal cancer treatment under near-infrared irradiation. Reprinted with permission from Ref. [51] (copyright 2019, Yu).

Chen et al. prepared nanoparticles loaded with DOX and used in synergy with Fe^3+^ for effective chemo-photothermal cancer therapy, drawing on the existing classical metal–phenolic network TA-Fe^3+^ nanoparticles for PTT, combined with the potent anticancer ability of DOX and EGCG combination therapy, in which DOX provides chemotherapeutic effects in a dual role and contributes to the realization of photothermal properties of nanoparticles (Figure 17). They synthesized nanoparticles (GTC) from EGCG and PEG-modified polyethyleneimine. Then DOX, an anti-cancer drug, was encapsulated in GTCs to prepare DOX@GTCs nanoparticles by electrostatic and p–p stacking interactions. The EGCG-Fe^3+^ network was successfully prepared after simple mixing with ferric chloride solution, which produced nanoparticles with photothermal capacity because the strongly coordinated phenol hydroxyl–metal charge transfer band exhibited strong absorption and high heat capacity in the near-infrared region. In vitro experiments showed that the prepared DOX@GTCs and DOX@GTCs–Fe nanostructures could be effectively internalized into HT-29 cells, and DOX@GTCs–Fe could also destroy these cancer cells by thermotherapy compared to DOX@GTCs. Animal experiments showed that tumors of both groups of mice injected with nanoparticles intravenously could be effectively suppressed with minimal side effects, confirming their accumulation at the tumor site. In addition, DOX@GTCs-Fe nanoparticles completely ablated the tumors under NIR irradiation. Thus, DOX@GTCs-Fe nanoparticles can achieve targeted drug delivery and have the ability to combine chemotherapy and photothermal therapy [51].

Bleomycin (BLM) is a class of glycopeptide antibiotics with strong antitumor activity widely used in the clinical treatment of squamous cell carcinoma and germ cell tumors [129]. However, BLM is dose-dependent and pulmonary toxic, causing lung lesions, and a rational drug delivery system needs to be developed to reduce toxicity. Due to a metal binding domain, BLM can bind to the transition metal Fe^2+^ to form BLM–metal complexes. Clinical studies have shown that the MPNs formed by EGCG and Fe^3+^ can effectively encapsulate BLM, transporting the drug to the tumor site. Since polyphenolic compounds cause strong adhesion to proteins in the blood, they can be coated with a layer of material to improve their stability [130,131], allowing for precise, effective treatment. Based on the above, Xie et al. developed a self-assembled drug delivery system that uses a bovine serum albumin (BSA)-modified Fe^3+^ metal–phenolic network to encapsulate BLM, thereby generating BFE@BSA and exploring the potential of BFE@BSA NPs as photothermal transduction agents and magnetic resonance imaging (MRI) contrast agents. MPNs composed of Fe^3+^ and EGCG have high photothermal conversion efficiency and can convert light energy into thermal energy under laser irradiation to locally increase tumor temperature and reduce damage to surrounding normal tissues, thus improving the therapeutic effect of PTT. In a synergistic, PTT/CDT/chemotherapy combination, the chemotherapeutic drug BLM is released to act on the tumor. Under laser irradiation, the Fe^3+^ metal–phenolic network converts light energy into heat energy, increasing free radicals to attack tumor cells and enhancing CDT through PTT [52]. Compared with the traditional treatment, encapsulating chemotherapeutic drugs with Fe-based MPN is more effective and stable. It has fewer toxic side effects, and Fe^3+^ can be used as a contrast agent, which has great potential for developing an integrated treatment system of contrast and treatment in the future.

### 4.3. Fe with ACN

Anthocyanins are plant pigments whose main sources are fruits and some cereals, legumes, and vegetables and whose main characteristics are unsaturated, water-soluble, and unoxidized flavonoid derivatives. The potential antitumor capacity of this compound has been demonstrated, and positive effects of anthocyanins have been reported for anti-inflammatory, antioxidant, anti-mutagenic, anti-proliferative, stimulation of autophagy or apoptosis, anti-invasive, and anti-metastatic properties [68].

Xu et al. reported a one-pot method for the preparation of multifunctional nanoparticles (FeAP NP) based on the coordination interaction of natural polyphenols (anthocyanins) extracted from fruits, Fe^III^ ions, and poly(L-glutamic acid)-methoxy poly(ethylene glycol) copolymers. The obtained FeAP NP exhibits good T1 relaxation and strong absorption in the NIR region, and thus, it can be used as a contrast agent for dual MR/PA imaging and as a photothermal agent for PTT. Besides, FeAP NP has the ability of dynamic decomposition in vivo, triggered by desferrioxamine mesylate (DFO), which disrupts the coordination between iron and ACN to achieve the effect of decomposing FeAP NP. Therefore, the application of DFO in disassembling FeAP NPs can modulate the hepatic clearance in vivo. This feature can address the challenges of EPR effects and renal clearance, as well as reduce the accumulation time of FeAP NPs in the liver. The final experiment concluded that FeAP NPs enabled complete tumor ablation in nude mice in vivo by PTT under the precise guidance of PA and MR imaging [35].

### 4.4. Fe with Qu

Quercetin (Qu) is a natural product widely distributed in vegetables, fruit peels, seeds, and beverages with abundant hydroxyl groups, and it is a polyphenol with excellent antioxidant capacity. Recently, natural polyphenols have been widely used to manufacture nanoparticles to facilitate the delivery of drugs, genes, and proteins. In contrast, quercetin’s ketones and hydroxyl groups provide three possible metal chelating sites that can coordinate with various metal ions [132]. Moreover, quercetin is also considered one of the most potent small molecule heat shock protein (Hsp) inhibitors, inhibiting the expression of Hsp70 by interfering with the phosphorylation of heat shock transcription factors to promote PTT efficacy. Therefore, nanodrugs made from quercetin (Qu) can be used directly as Hsp inhibitors and as a backbone component of PTT materials, thus avoiding the problem of drug loading and release.

On the other hand, the hydroxyl group of quercetin can be converted to ketones when scavenging free radicals (e.g., O^2-^-, -OH, -OR, etc.). This may weaken the metal chelating ability of quercetin, providing an opportunity for in vivo decomposition and elimination of Qu–MP nanoparticles. At the same time, ROS scavenging ability could reduce ROS-related inflammation. Afterward, free quercetin can continue to exert its inherent anticancer, anti-inflammatory, antiviral, and antibacterial pharmacological activities, further contributing to synergistic anti-inflammatory and antitumor therapy [70].

Based on the above advantages of quercetin, Yang et al. newly developed a Qu–MP nanodrug, so quercetin is not only a heat shock protein (Hsp70) inhibitor but also a backbone of Qu-Fe^II^, which achieved complete ablation of tumors by NIR light-induced low-temperature PTT (45 °C) without heat stress to normal tissues. Due to the ability of quercetin to scavenge ROS, Qu-Fe^II^ effectively reduced intracellular ROS and inflammatory factor levels in vivo. At the same time, quercetin–iron coordination was attenuated in scavenging ROS, which triggered the breakdown of Qu-Fe^II^, resulting in adequate clearance of nanoparticles from major organs 168 h after intravenous injection. In addition, Qu-Fe^II^ has dual photoacoustic and magnetic resonance imaging capabilities, providing excellent spatial resolution and imaging depth for accurate tumor diagnosis and for monitoring nanodrug catabolism in vivo. Thus, Qu-Fe^II^ combines precise diagnosis, superior cryo–PTT efficacy, ROS elimination and anti-inflammatory effects, dynamic catabolism, and renal clearance integrated into a single nanodrug, which is very promising for future clinical cancer treatment [53].

### 4.5. Fe with EA

Ellagic acid is a chromium-diketone derivative that acts, mainly, as an antioxidant in vivo, and EA plays a vital role in the anti-atherogenic, anti-inflammatory, and neuroprotective effects [69]. The structure, with four hydroxyl groups and two lactone hydrophilic parts, can scavenge many reactive oxygen species [133]. At physiological pH, EA inactivates •OH, peroxyl radicals (ROO⋅), etc. [134,135], and this anti-free radical property does not decrease during metabolism. EA can also chelate with Fe^2+^, and the deprotonated EA can also chelate with Cu, where the metal ions accelerate the oxidation process. Still, through chelation, EA combines with metal ions to form a “preventive” antioxidant that does not promote oxidation. In addition to being an antioxidant, Saha et al. demonstrated that catechol on EA could chelate with Fe^3+^, giving it a good photothermal effect, and that iron ions have good T2 MR imaging capabilities, which can aid in diagnosis and treatment [136].

The Fe–EA framework prepared by Zhao et al. has good photothermal conversion ability, converting light energy into heat energy under laser irradiation to locally heat the tumor site and kill tumor cells, minimizing the toxic effects on healthy cells. The team found that the MPN nanoparticles were not significantly cytotoxic in vivo, and they were biodegradable under the tumor microenvironment conditions, after several months of oral administration in mice, without showing toxicity [36]. Based on the construction of the MPN drug delivery system, whether the Fe-EA can be loaded with photothermal agents with a better photothermal conversion rate or drugs that can stop the growth of cancer cells is a promising research direction. The development of the Fe–EA combined therapeutic drug delivery system is an area that still needs to be explored.

### 4.6. Fe with PEG-Polyphenol

Many drugs with efficient cancer therapeutic effects are being developed, but most have limitations: biocompatibility, possibly stability, and possibly bio toxicity. Surface modification is a convenient and effective way to do just that. PEG is one of the most successful choices for modification [137]. Amphiphilic PEGs have good water solubility, biological inertness, and non-toxicity, and they are widely used in the pharmacology of proteins and drug carriers [72]. Moreover, PEGylated liposomes are considered invisible liposomes that significantly prolong the circulation time of nanocarriers, reduce the interaction with plasma proteins, and decrease the uptake by the reticuloendothelial system [138]. Liposomes coated with PEG also improve the accumulation of nanocarriers in tumor tissues by exerting the EPR effect [139]. Besides, PEG-polyphenol has abundant phenolic hydroxyl groups that can chelate with metals, thus its potential as an organic ligand for metal–phenolic networks.

Wei et al. then used PEG-polyphenol to confer the ability to evade recognition by the immune system and to prolong the drug’s circulating half-life. They have secondly used the encapsulation properties of PEG-polyphenol to prevent drug spillage and to release the drug once these particles were taken up by the target cells to achieve its function. They loaded folic acid (FA)-doped capsules with hematoporphyrin monomethyl ether (HMME), a water-soluble photosensitizer, to obtain HMME-doped PEG-MPN capsules (MPN@HMMEs). This strategy emphasizes enhancing the PDT effect, which improves the targeting ability of photosensitizers to cancer cells and allows normal cells to survive after PDT. With the assistance of FA, MPN@HMMEs are taken up by cancer cells through the folate receptor (FR) pathway.

Meanwhile, MPN@HMMEs resist normal cells due to their low cell association properties and FR expression. After FR-mediated endocytosis, the capsule is transferred to the endo nucleosome and, then, dissociated in the lysosome due to the physiologically relevant acidic pH environment. As a result, the encapsulated photosensitizer is released from the lysosome into the cytoplasm. Under 638 nm laser irradiation, the ROS generated by the photosensitizer induces apoptosis [21].

**Figure 18:**
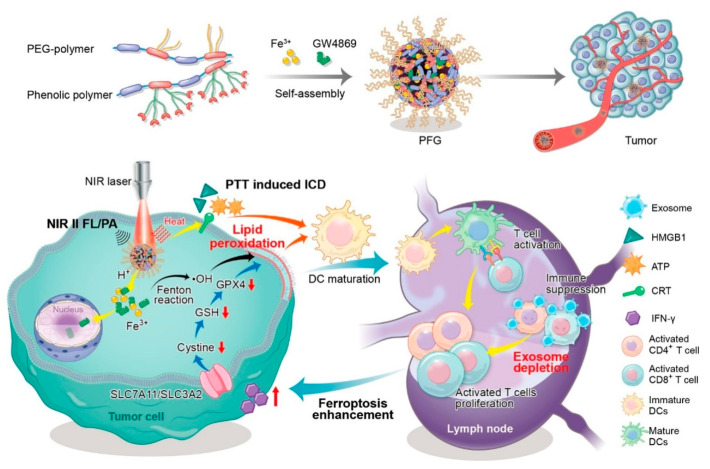
Schematic illustrations of PFG MPNs for phototheranostic effect, relief of exosomal immunosuppression, ferroptosis enhancement, and immune stimulation. A self-assembly process for the preparation of PFG MPNs was first presented. After intravenous injection, PFG MPNs could generate heat under laser irradiation, achieving PTT-augmented ICD by using a precise NIR II FL/PA imaging track. Meanwhile, released GW4869 from PFG MPNs strongly induced exosome depletion, resulting in antitumor immune activation against tumor cells. Additionally, the reactivated T cell proliferated and secreted high levels of IFN-γ to downregulate ferroptosis-relevant molecules (including SLC7A11, SLC3A2, cystine, GSH, and GPX4), enhancing the ferroptosis induced by rich Fe^3+^ released from PFG MPNs. Reprinted with permission from Ref. [56] (copyright 2022, Xie).

Xie et al. make more use of PEG-polyphenol to modify the material itself. They fit good ferroptosis inducers in nanosystems with phenolic groups on phenolic polymers and add hydrophilic polyethylene glycol polymers to functionalize nanoparticles to be amphiphilic, forming polymer-encapsulated Fe^3+^ MPNs (PF MPNs). These amphiphilic semiconductor polymers serve as good structural motifs for metal–polyphenol coordination to effectively form MPNs carrying therapeutic drugs with unique properties (e.g., fast coordination process, excellent metal chelating ability, and pH responsiveness). They designed phototherapeutic metal–phenolic networks (PFG MPNs), mainly, by encapsulating a semiconductor polymer assembly of an iron exfoliation inducer (Fe^3+^) and exosome inhibitor (GW4869). The metal networks have a good photothermal effect, and PTT can promote immunogenic cell death, thus alleviating the silencing of exosomes during DC maturation. GW4869-mediated PD-L1 exosome inhibition activated T cells and enhanced ferroptosis, causing PFG MPNs to significantly impair exosome inhibition of DC maturation and T cell function (Figure 18). The prominent role played by the MPN in this system is to quasi-convert light energy into heat energy, enhancing PTT and, thus, driving immunogenic cellular death [56].

### 4.7. Fe with PEG-Ce6-Polyphenol and Gossypol

**Figure 19:**
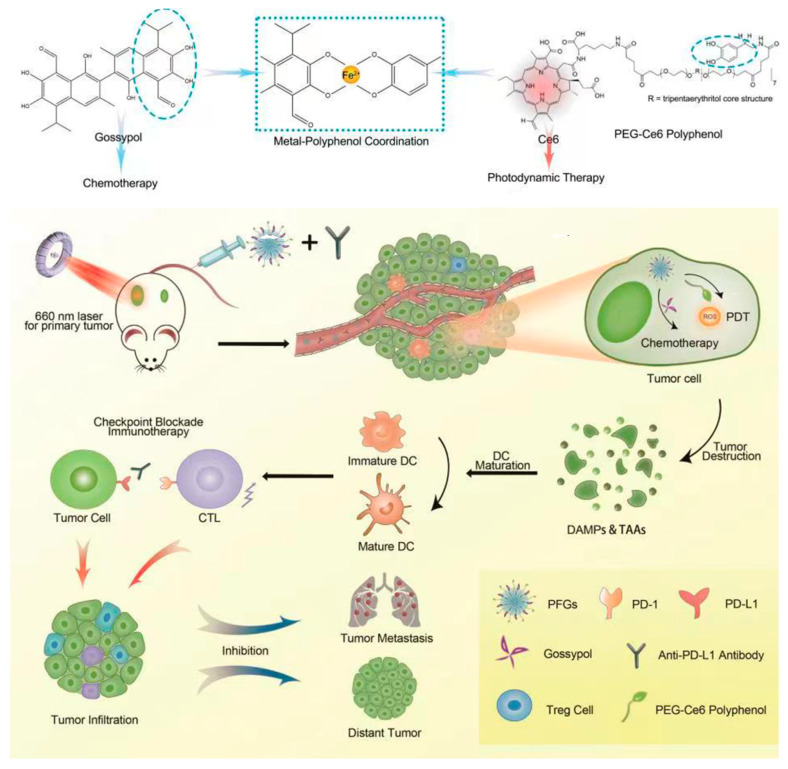
Schematic illustrations of the fabrication process of PFGs. PFGs combined with a PD-L1 checkpoint blockade for immune response enhancement and inhibiting tumor proliferation and metastasis. DAMPs: damage-associated molecular patterns. TAAs: tumor-associated antigens. Reprinted with permission from Ref. [39] (copyright 2021, Zhang).

Immunogenic cancer cell death (ICD) elicits an antitumor immune response and plays a key role in reversing the immunosuppressive system and prolonging antitumor immunity. It has been shown that using PDT, combined with chemotherapy, can enhance the level of ICD [39]. Gossypol is an ideal polyphenol ligand for forming MPN due to its anticancer properties and lack of significant systemic side effects. Chlorin e6 has been developed as a good photosensitizer for the application of PDT to kill tumor cells. Inspired by this, Zhang et al. combined the photosensitizer Ce6 and PEG-polyphenols to form PEG-Ce6-polyphenols. They established a metal–polyphenol nanocomplex (PFGs) with the coupling of Fe^2+^ and the insertion of gossypol (Figure 19). The nanocomplex is a composite MPN with the gossypol fraction effectively acting as a chemotherapeutic agent and Ce6 acting as a photosensitizer of the system to induce the Fenton reaction for PDT. In this study, PFGs in the form of nanoparticles were effectively accumulated at the tumor site after injection from the vein. Effective chemotherapy/PDT-induced ICD performance was demonstrated under 660 nm laser irradiation. The experimental results showed that PFGs + laser combined with anti-programmed cell death ligand-1 (PD-L1) antibodies enhanced the infiltration of cytotoxic T lymphocytes (CTLs) in tumor tissues and increased the secretion of immune-related cytokines to counter tumors [39]. This method successfully realized the combination of chemotherapy and PDT, and it enhanced the immune system through checkpoint/blockade immunotherapy (IT).

## 5. Fe-Based MPNs in Other Therapies

Though CDT and PT are the two most commonly used tumor treatments for Fe-based MPNs materials, a small percentage of Fe-based MPNs can also play a key role in other therapies. Some of them are radiotherapy, chemotherapy alone, and immunogenic cell therapy in a synergistic setting.

### 5.1. Fe with TA

**Figure 20:**
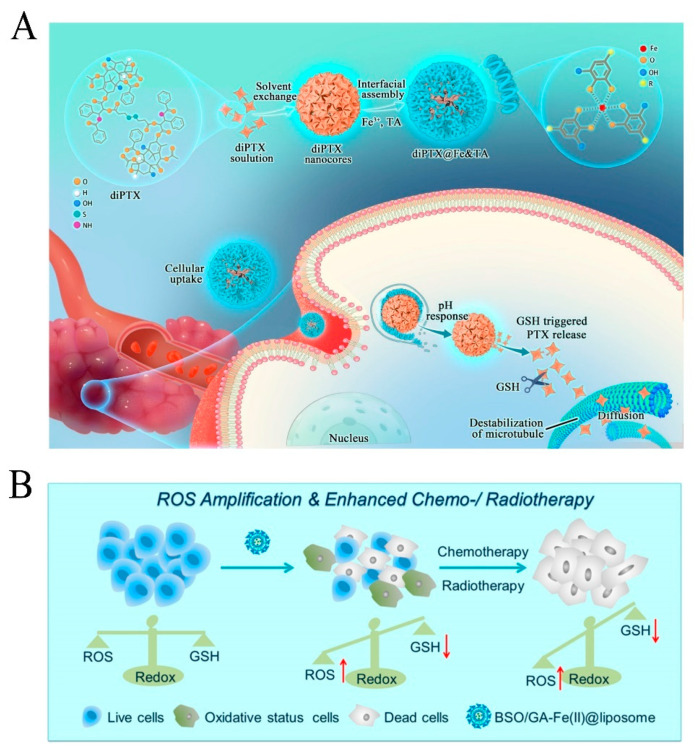
(**A**) The procedure for fabricating diPTX@Fe&TA nanoparticles, as well as intracellular pH and redox-triggered programmed drug release. Reprinted with permission from Ref. [48] (copyright 2022, Yi). (**B**) ROS-mediated chemo-/radiotherapy sensitization. Schematic illustration of the efficient disruption of redox homeostasis in the tumor by BSO/GA–Fe(II)@liposome for enhanced chemo/radiotherapy. Reprinted with permission from Ref. [33] (copyright 2019, Dong).

It is not uncommon for MPNs to optimize chemotherapy, but there are few cases of optimizing chemotherapy alone. Representative among the Fe-based MPNs is a nanoparticle (diPTX@Fe & TA) consisting of TA-Fe^3+^ coating wrapped around the chemotherapeutic drug dimeric paclitaxel (diPTX) designed by Yi et al. (Figure 20A). The method uses MPN formed by Fe^3+^ and TA, which overcomes the drawbacks of conventional nano-delivery systems and is a preparation method with adequate drug loading capacity and superior stability. Under the good pH responsiveness of MPN, the conversion of the prodrug diPTX into free paclitaxel (PTX) was achieved by the high concentration of intracellular GSH, which prompted the rapid diffusion of free PTX to microtubules and disrupted the balance between microtubule polymerization and depolymerization, ultimately leading to apoptosis of the tumor cells [48]. After a simple MPN encapsulation of the prodrug, the drug delivery and stability were substantially improved, and the toxicity to organs was negligible, achieving a good chemotherapeutic effect. However, chemotherapy is inevitably more limited in inhibiting tumor spread at the source. The therapeutic scope of optimizing chemotherapy is limited, and there is still much potential for improvement.

It has been shown that highly aggressive triple-negative breast cancer (TNBC) is prone to ferroptosis [140]. Unlike apoptosis, tumor cells with ferroptosis promote the activation and maturation of bone marrow-derived dendritic cells and stimulate immune responses against tumors by releasing damage-associated molecular patterns (DAMPs) in vivo. Thus, immunotherapy combined with ferroptosis may synergistically lead to the suppression and eradication of tumor cells. Previous studies have concentrated on developing various Fe-based nanomaterials, while the role of ferroptosis in the induction of apoptosis by tumor cells has remained unsatisfactory. Subsequently, it was reported that glutathione peroxidase4 (GPX4) could maintain the homeostasis of the membrane lipid bilayer by depleting GSH, which converts LPO to non-toxic lipid alcohols, resulting in inefficient ferroptosis in tumor cells. Thus, it is seen that simultaneous upregulation of ROS and inactivation of GPX4 are significant for the treatment of ferroptosis [141].

Inspired by this, Yang et al. constructed a rigid metal-polyphenolic shell-modified nanocarrier (ssPPE_Lap_@Fe-TA) for enhanced ferroptosis. The exterior is coated with a Fe^3+^–TA network that can be disintegrated in acid to drive an effective Fe^3+^/Fe^2+^ conversion and induce ferroptosis in tumor cells. With its disulfide-containing polyphosphoester (ssPPE) release, GPX4 is inactivated by the disulfide–thiol exchange reaction, depleting intracellular GSH and triggering the rapid release of H_2_O_2_ inducer (β-lapachone) as a way to enhance intracellular H_2_O_2_ levels significantly. Ultimately, the synergistic effect of an increased Fe^3+^/H_2_O_2_ supply and GSH depletion significantly induces ferroptosis in tumor cells, resulting in effective tumor suppression. Additionally, the amplified ferroptosis induced tumor cell death in an immunogenic manner, thereby promoting dendritic cell (DCs) maturation and T cell infiltration. Combined with a-PD-L1, ssPPE_Lap_@Fe-TA dramatically inhibited the 4T1 tumor growth and lung metastasis [49]. Not surprisingly, the therapeutic mechanism of using immunotherapy in synergy with cellular ferroptosis is another innovative strategy for applying Fe-based MPNs.

### 5.2. Fe with GA

Radiotherapy (RT) is the use of various rays (X, γ, α, e, proton, neutron, heavy ion, etc.) to interrupt DNA strands in the nucleus (called direct action) or ionize water to produce ions to interrupt DNA strands again (called indirect action). DNA strands break, and cells cannot divide and proliferate without restriction, resulting in the inhibition or killing of tumors [142,143]. GA has a strong ability to scavenge free radicals, possess antioxidant activity, and exhibit pro-oxidant behavior in Fenton chemistry [62]. Dong et al. used a strategy to amplify intracellular oxidative stress and prepared a unique liposome-based ultrasmall nanocomplex (BSO/GA–Fe(II)@liposome). The nanocomplex, with the help of MPN (GA-Fe^2+^) formed by the coordination of GA with Fe^2+^, can act as a catalyst for the Fenton reaction by directly supplying highly catalytically active Fe^2+^, enabling the sustained conversion of H_2_O_2_ to the highly cytotoxic •OH. Since buthionine sulfoxide (BSO) effectively induces the depletion of intracellular GSH, the simultaneous use of BSO and GA-Fe2+ can amplify intracellular oxidative stress through effective ROS production and an accompanying lack of GSH, which confers more effective killing of cancer cells. The superior metal ion chelating ability of GA was utilized, and after labeling with the radioisotope ^99m^Tc^4+^, it was able to facilitate the in vivo tracking. Finally, the prepared liposomes were used to encapsulate BSO and GA-Fe^2+^ to obtain nanocomplexes for the synergistic treatment of RT and chemotherapy (Figure 20B). The Fe^2+^ released from GA-Fe^2+^ was oxidized to Fe^3+^ and, then, reduced to Fe^2+^ by GA to provide sustainable catalytic H_2_O_2_ activity. The GA-Fe^2+^-mediated production of •OH and BSO-mediated depletion of GSH significantly enhance the oxidative stress within the tumor and achieve effective therapeutic effects of RT and chemotherapy [33].

## 6. Conclusions and Outlook

From the current perspective, metal–phenolic networks (MPNs) play an irreplaceable role in tumor treatment. The characteristics show that MPNs have high-quality features such as being green, non-toxic, and sensitive pH responsiveness, as well as exhibiting good photothermal performance, good tunability, and good biocompatibility. Fe ions are the most commonly used metal ion ligands when forming MPNs, and three of the most widely used polyphenol ligands are available. The combination of TA with Fe^3+^ is often used as a nanocoating to encapsulate drugs for effective targeting therapy. Combining EGCG with Fe^3+^/Fe^2+^ can be used as nanoparticles co-encapsulated with medicines, and they act as reaction catalysts when released. PEG-polyphenols with Fe^3+^/Fe^2+^ can be combined with other polyphenols to form hybrid MPNs that can significantly prolong the nanocarrier blood circulation time.

Though the Fe-based MPN is just a small subclass of the MPNs family, the optimal design and application strategies of various Fe-based MPNs have been somewhat developed in recent years. Compared with other nanomaterials, the faster synthesis rate and higher tunability have greatly improved tumor treatment. The applications in CDT and PT have been investigated more, and the materials modified with Fe-based MPNs can achieve significant breakthroughs in synergistic therapy. However, the research of Fe-based MPNs in tumor treatment is still in its infancy. Currently, several shortcomings and challenges are still waiting to be addressed further. (I) Among other therapies, such as starvation therapy and immunotherapy, few instances exist of using Fe-based MPNs, alone, to improve therapeutic efficiency. These are combined with other copolymer compounds to form hybrid coatings to improve biocompatibility and therapeutic efficiency. Many Fe-based MPNs are therapeutically monotonous, making it difficult to achieve therapeutic improvement without synergistic treatment and chemotherapeutic drugs. (II) Even though PEG-polyphenols have been developed for Fe-based MPNs, the reduction in delivery efficiency will directly lead to a diminished therapeutic effect for the accumulation at the tumor site through the EPR effect, which is insufficient to achieve high tumor selectivity. While the Pt prodrug polyphenols have better tumor inhibition than free Pt prodrugs and cisplatin, they are less responsive to pH. The release effect at TME is not fully reflected. Therefore, more versatile drug delivery systems for Fe-based MPNs should be developed.

The family of MPNs is very abundant. Nevertheless, the number of Fe-based MPNs currently used in tumor therapeutic research is relatively small. Natural polyphenols, such as Gossypol, GA, ACN, EA, and Qu, have much greater potential to be explored. Based on their properties, they are very suitable to be combined with drugs to develop new nanoplatforms. Notably, using Fe-based MPNs for synergistic therapy will become a significant development trend which can not only exploit the generic properties of MPNs but also improve the tumor efficacy with the chemotherapeutic drugs or copolymer complexes. Hybrid polyphenolic networks based on Fe^3+^/Fe^2+^ will also be a new design idea to combine natural polyphenols with synthetic polyphenols to optimize the deficiency for tumor treatment and improve the in vivo compatibility. In summary, despite the many problems, Fe-based MPNs have considerable potential for development in biomedical applications. Finally, we hope that this paper’s overview and evaluation of Fe-based MPNs applications will inspire more interesting and meaningful studies.

## Figures and Tables

**Table 1:** Fe-based MPNs used polyphenols in tumor treatments.

Polyphenol	Structure	Application
TA	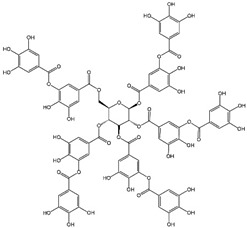	CDT/PTT/PDT/chemotherapy/IT
GA	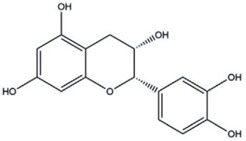	RT/chemotherapy
EGCG	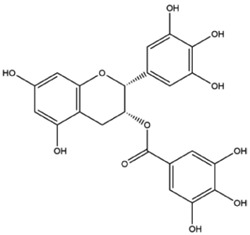	CDT/PTT/chemotherapy
ACN	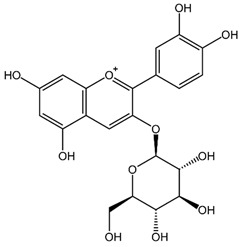	PTT
EA	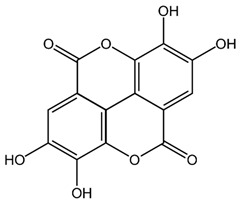	PTT
Qu	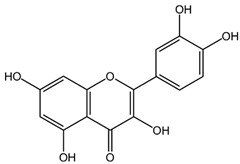	PTT
Gossypol	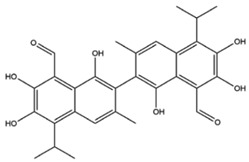	IT/chemotherapy
PEG-polyphenol	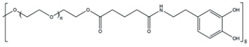	CDT/PTT/chemotherapy
PEG-Ce6-polyphenol	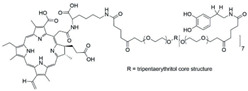	IT/PDT
Pt prodrug-polyphenol	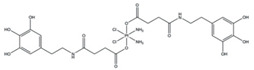	chemotherapy

**Table 2:** Overview of selected Fe-based MPNs used for various tumor treatments.

Polyphenol	MPN Formation	Materials	Mechanism	Ref.
TA	Nanocoating	Den-DOX-TA-Fe^3+^	Induces ferroptosis and enhances ROS.	[40]
	Nanocoating	CaO_2_@ZIF8@MPN	Enhances Fenton reaction of Ca^2+^ react with H_2_O_2_.	[41]
	Nanocoating	Au@MPN	Self-supplies H_2_O_2_ and autocatalytic Fenton reaction.	[42]
	Nanocoating	G5.NHAc-Toy@TF	Upregulates ROS, downregulates GSH and GPX-4, and allows LPO to accumulate in cells, leading to ferroptosis.	[43]
	Nanocoating	HSA-GOx-TPZ-Fe^3+^-TA (HGTFT)	Accelerates Fe^3+^/Fe^2+^ conversion, starvation therapy.	[44]
	Nanocoating	PID@Fe-TA	pH-responsiveness and NIR enhance ROS.	[45]
	Nanocoating of TA-Fe^3+^/Mn^2+^	DZ@TFM	Induced ferroptosis and Mn^2+^-activate DA inhibit PD-L1.	[46]
	Nanocoating	SRF@Fe^III^TA-NAPP	Release of SRF induces ferroptosis while enhancing Fenton response and ROS level	[47]
	Nanocoating	diPTX@Fe&TA	Convert diPTX into free PTX to diffuse to microtubules, disrupting the microtubule balance and leading to tumor cell apoptosis.	[48]
	Nanocoating	ssPPE_Lap_@Fe-TA	Increased Fe^3+^/H_2_O_2_ supply and GSH depletion, immunotherapy synergistic cellular ferroptosis	[49]
GA	Nanoparticle	BSO/GA-Fe(II)@liposome	Generation of ROS and simultaneous consumption of GSH to co-amplify intracellular oxidative stress.	[33]
EGCG	Nanocoating of EGCG and PVP with Fe^2+^	FeEP-NPs	Continuously supplies Fe^2+^ with mildly high temperature to promote Fenton reaction.	[50]
	Nanocomposite	DOX@GTCs-Fe	Synergistic treatment with PTT and chemotherapy in the presence of NIR.	[51]
	Nanocoating	BFE@BSA NPs	BLM improves local temperature to enhance Fenton response and ROS level.	[52]
ACN	Nanocomposite	FeAP-NPs	EPR effect, DFO-triggered in vivo dynamic disassembly	[35]
EA	Nanoparticle	Fe-EA	pH-activated amplification of MRI contrast effect and in vivo PTT for tumor ablation	[36]
Qu	Nanocomposite	Qu-Fe^II^P	EPR effect, inhibition of Hsp70 expression, ablation and elimination of tumors by low temperature PTT	[53]
Pt prodrug-polyphenol	Nanocomposite	PTCG NPs	Cascade reaction enhances H_2_O_2_ levels and catalyzes the production of toxic ROS.	[54]
PEG-polyphenol	Nanocoating/Nanocomposite	CADNs	GOx catalyzes the achievement of starvation therapy, killing tumor cells through Fe^3+^/Fe^2+^-mediated Fenton reaction in conjunction with chemotherapy.	[55]
	Nanoparticle/Nanocomposite	PFG MPNs	Relief of exosomal immunosuppression, ferroptosis enhancement, and immune stimulation.	[56]
	Nanocomposite	MPN@HMME	HMME, as a photosensitizer, dissociated in lysosomes and killed carcinoma cells with a laser.	[21]
Gossypol PEG-Ce6-polyphenol	Nanocomposite	PFGs	Ce6, as a photosensitizer induces a Fenton reaction for PDT to enhance the level of ICD.	[39]

## Data Availability

Not applicable.
